# An Essential Nonredundant Role for Mycobacterial DnaK in Native Protein Folding

**DOI:** 10.1371/journal.pgen.1004516

**Published:** 2014-07-24

**Authors:** Allison Fay, Michael S. Glickman

**Affiliations:** 1 Immunology Program, Sloan Kettering Institute, Memorial Sloan Kettering Cancer Center, New York, New York, United States of America; 2 Division of Infectious Diseases, Memorial Sloan Kettering Cancer Center, New York, New York, United States of America; University of Geneva Medical School, Switzerland

## Abstract

Protein chaperones are essential in all domains of life to prevent and resolve protein misfolding during translation and proteotoxic stress. HSP70 family chaperones, including *E. coli* DnaK, function in stress induced protein refolding and degradation, but are dispensable for cellular viability due to redundant chaperone systems that prevent global nascent peptide insolubility. However, the function of HSP70 chaperones in mycobacteria, a genus that includes multiple human pathogens, has not been examined. We find that mycobacterial DnaK is essential for cell growth and required for native protein folding in *Mycobacterium smegmatis*. Loss of DnaK is accompanied by proteotoxic collapse characterized by the accumulation of insoluble newly synthesized proteins. DnaK is required for solubility of large multimodular lipid synthases, including the essential lipid synthase FASI, and DnaK loss is accompanied by disruption of membrane structure and increased cell permeability. Trigger Factor is nonessential and has a minor role in native protein folding that is only evident in the absence of DnaK. In unstressed cells, DnaK localizes to multiple, dynamic foci, but relocalizes to focal protein aggregates during stationary phase or upon expression of aggregating peptides. Mycobacterial cells restart cell growth after proteotoxic stress by isolating persistent DnaK containing protein aggregates away from daughter cells. These results reveal unanticipated essential nonredunant roles for mycobacterial DnaK in mycobacteria and indicate that DnaK defines a unique susceptibility point in the mycobacterial proteostasis network.

## Author Summary

All living organisms use protein chaperones to prevent proteins from becoming insoluble either spontaneously or during cellular stress that can damage proteins. The HSP70 chaperone DnaK has been well characterized in *E. coli* and is important for that bacterium to resist protein denaturation from heat, but is dispensable for cell growth in the absence of stress due to redundancy with other chaperone systems. However, the function of chaperones in bacterial pathogens, which are exposed to protein stress within the host, has received less attention. Here we examine the function of DnaK in mycobacteria, a genus that includes multiple human pathogens, and find that DnaK is required for cell growth. This essential function is due to a lack of redundancy with other chaperone systems for the folding of proteins, even in the absence of stress. These findings expand the paradigm of DnaK function and identify DnaK as a promising target for antibiotic development for mycobacteria.

## Introduction

Proper protein folding is essential for all organisms and assures that the primary sequence of the polypeptide forms its functional tertiary and quaternary structures. Protein chaperones are present in all domains of life and serve multiple functions in protein homeostasis. During translation, chaperones are required to assure proper protein folding and prevent protein aggregation, which can occur as hydrophobic segments of the protein emerge from the ribosome. After synthesis, protein denaturation is a common event due to exogenous proteotoxic stresses such as heat and oxidation, correction of which requires chaperone systems to refold denatured proteins when possible, and facilitate disaggregation and degradation when refolding is not possible. The importance of chaperone function for cellular viability is reflected in the frequent redundancy between chaperones for protein folding and aggregate processing [1]–[4].

The Hsp70 family of chaperones is widely distributed in both prokaryotic and eukaryotic cells [5]. The best studied Hsp70 chaperone of bacteria is *E. coli* DnaK. DnaK is a central hub for protein folding, shuttling misfolded peptides to other chaperones and proteases for resolution, a function that is essential during the protein denaturation that occurs during heat shock [6]–[9]. In addition to its effector function in the heat shock response, DnaK also regulates this response by destabilizing the alternative sigma factor, σ^32^, preventing aberrant induction of the heat shock response during non-stress conditions and turning off the response after heat shock [10]. However, In *E. coli*, DnaK is nonessential for native protein folding because of redundancy with Trigger Factor, which associates with proteins soon after emergence from the ribosome [11]. Although *dnaK*/*tf* double mutants are nonviable, overexpression of GroEL, SecB, or Hsp33 can suppress the synthetic lethality of *dnaK/tig* double mutants [10], [12]–[16]. Furthermore, examination of proteins that interact with DnaK indicates that most client proteins that require DnaK for proper folding and/or stability are largely non-essential, suggesting that loss of function of these proteins in the absence of DnaK does not impact viability [7]. However, in the absence of both DnaK and TF, the *E. coli* cell suffers proteostasis collapse characterized by global insolubility of nascent proteins [7].

In bacteria other than *E. coli*, the function of DnaK has not been studied extensively. In mycobacteria, DnaK regulates the heat shock response through its interaction with the HspR C terminal tail, which becomes insoluble upon heat shock, thereby relieving repression of chaperone genes [17]. The mycobacterial heat shock response is negatively regulated by the repressors HspR and HscA; and positively through σ^H^ [18], [19]. Deletion of *hspR*, which derepresses several chaperones involved in the heat shock response, including ClpB, alpha-crystalin, and DnaK-GrpE-DnaJ1, led to decreased persistence after the initial phase of infection in a mouse model suggesting that *dnaK* and other HspR regulated genes must be controlled during infection for optimal growth and persistence [19]. Host inflicted proteotoxic stress is likely a significant *in vivo* stress for *M. tuberculosis* during infection, yet the function of the mycobacterial chaperone network in native and stress induced proteostasis is incompletely understood. Additionally, Mtb DnaK is found in culture filtrates [20], [21] and on the bacterial surface [22], and has a role in pathogenesis by modulating host immune responses [22]–[24]. Despite substantial progress in targeting chaperone function in malignant human cells [25], [26], inhibition of chaperone function as an antimicrobial strategy is relatively unexplored, in part because of the redundancy of the chaperone network. Using a model mycobacterial species, *M. smegmatis*, we characterized the function of DnaK in mycobacteria and find an unanticipated lack of redundancy that places mycobacterial DnaK as a central chaperone in both native and stress induced protein folding.

## Results

### DnaK is essential for growth of *M. smegmatis*

To study the function of mycobacterial DnaK, we attempted to delete *dnaK* from the *M. smegmatis* chromosome. Initial attempts yielded no allelic replacements, suggesting that DnaK may be essential for viability. Provision of a second copy of *dnaK* at the *attB* phage integration site allowed replacement of the chromosomal *dnaK* with an unmarked Δ*dnaK* allele lacking the first 1765 bp of the 1869 bp *dnaK* ORF (Figure S1A). We then attempted to remove the second copy of *dnaK* from *attB* by marker exchange with either a vector, pMV306kan, or a plasmid encoding DnaK and conferring kanamycin resistance, pAJF447. Only transformation with pAJF447 yielded transformants that were kanamycin resistant at 30°C or 37°C (Figure S2A). We observed small numbers of kanamycin resistant transformants in vector transformed cells, but these cells continued to express DnaK, indicating that the second copy of *dnaK* was not lost in these transformants (Figure S2A). This failure to remove the copy of *dnaK* from *attB* in our Δ*dnaK* strain suggested that *dnaK* was required for growth at 30°C (low) and 37°C (high) temperatures. We observed a similar essentiality for the DnaK cofactor GrpE. After constructing a strain with a *grpE* deletion and a second copy of *grpE* at *attB* (MGM6023; Figure S1B) we were unable to remove this second copy of *grpE* from *attB* by marker exchange at either 30°C and 37°C (Figure S2B), demonstrating that both DnaK and GrpE are required for growth of *M. smegmatis*. Similar results were obtained when we attempted to delete the entire *dnaK* operon suggesting that all three components of the operon are essential (data not shown).

**Figure 1:**
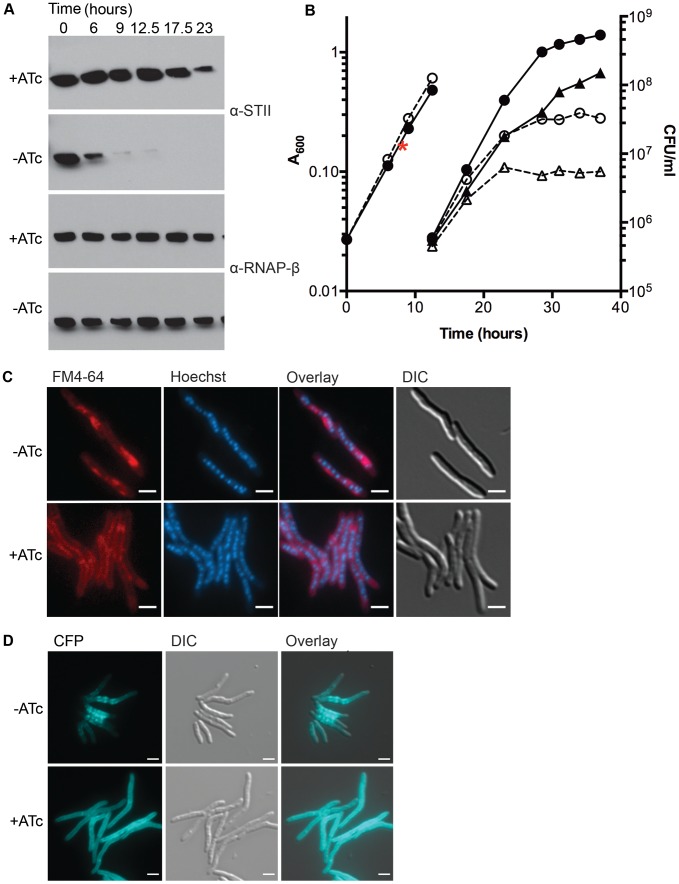
Depletion of DnaK is bacteriostatic in *M. smegmatis* and disrupts membrane structure. (A) Depletion of DnaK. Immunoblots of lysates prepared from DnaK-STII depletion cultures after withdrawal of the ATc inducer. Top panel is blotted for StrepTagII, bottom for RNAP-β. (B) Growth and survival of DnaK replete and depleted cells. Shown are optical density (left axis, circles) and CFU (right axis, triangles) of Tet-DnaK (MGM6005) grown with (solid lines, filled symbols) or without (dashed lines, open symbols) ATc. At 12.5 hours, cultures were diluted to maintain logarithmic growth. CFU/ml was determined for timepoints post dilution. The red asterisk marks the timepoint at which DnaK-STII was undetectable by immunoblot. (C) Loss of DnaK perturbs membrane structure but not nucleoid or cell morphology. MGM6005 grown with or without ATc for 16 hours. Cells stained with FM 4-64 and Hoechst prior to imaging. (D) DnaK is required for proper membrane protein localization. A Tet-DnaK strain expressing a membrane localized 3X-mCerulean (MGM6015) observed with (+ATc) or without (−ATc) DnaK for 16 hours. White bars indicate 2 µm. Exposure times were FM 4-64 500 ms, DAPI 500 ms and CFP 500 ms.

To further study the function of DnaK, we generated a depletion strain, MGM6005, which encodes an anhydrotetracycline (ATc) inducible allele of DnaK with a C terminal StrepTagII (STII). By 9 hours after withdrawal of ATc, DnaK-STII was undetectable by immunoblot with anti-STII antibodies (Figure 1A). Cells lacking DnaK continued to grow for an additional 12 to 15 hours without detectable DnaK, at which point optical density stabilized, in contrast to the continued replication of DnaK replete cells (Figure 1B). The viability of growth arrested cells lacking DnaK was determined by culturing dilutions onto selective media with ATc for both depletion and control cultures. The number of viable cells remained constant in the growth arrested, DnaK depleted population, indicating that loss of DnaK is bacteriostatic rather than bactericidal over the time course of the experiment (Figure 1B). The DnaK chaperone system has been previously implicated in protection and recovery of bacterial cells from heat shock [10], [27]–[29]. In *M. tuberculosis*, loss of σ^H^ has been shown to result in decreased survival at 53°C, a phenotype that was attributed to attenuation of the mycobacterial heat shock response, including the induction of DnaK [18]. To directly test the contribution of DnaK to heat shock response, we depleted DnaK for 12 hours prior to measuring cell viability at 53°C. Cells lacking DnaK were 100 fold more sensitive to killing by heat compared to DnaK replete cells (Figure S3).

To examine the morphologic correlates of the growth arrest that accompany DnaK depletion, we examined DnaK depleted and control cells by microscopy. Nucleoid morphology (Figure 1C, Hoechst panel) and cellular morphology (Figure 1C, DIC panel) of DnaK depleted cells were indistinguishable from that observed in wildtype cells, suggesting that DnaK depletion did not alter cell gross morphology. However, membrane staining by the lipophilic dye FM 4-64 was altered in cells lacking DnaK. In contrast to the homogenous distribution of the FM 4-64 membrane staining pattern seen in wild type cells, FM 4-64 in DnaK depleted cells was no longer evenly distributed along the entire periphery of the cell, but rather accumulated in a patchy pattern at midcell (Figure 1C). To examine whether this change in membrane staining pattern was accompanied by changes in membrane protein localization, we fused *E. coli* MalF transmembrane domains 1 and 2 to three copies of mCerulean. This fusion was evenly distributed in the membrane of cells expressing DnaK (Figure 1D, bottom). In contrast, upon DnaK depletion, MalF-mCerulean accumulated at midcell (Figure 1D, top) in a pattern that colocalized with FM 4-64 in the absence of DnaK (Figure S4). Taken together, these results indicate that loss of DnaK affects membrane structure and/or membrane protein localization with relative preservation of overall cell dimensions and nucleoid morphology.

To assess whether the membrane alterations seen with loss of DnaK affected cell permeability, we utilized a previously described assay for measuring ethidium bromide (EtBr) permeability [30]–[32]. DnaK depleted cells showed increased accumulation of EtBr as compared to replete cells (Figure S5). The addition of carbonyl cyanide 3-chlorophenylhydrazone (CCCP), an efflux inhibitor shown to increase EtBr accumulation [31], led to an increase of EtBr accumulation in both DnaK depleted and replete cells, indicating that DnaK depleted cells were still capable of EtBr efflux. Cells lacking DnaK still had increased accumulation relative to replete cells in the presence of CCCP (Figure S5) indicating that the increase in EtBr in DnaK depleted cells was due to an increase in cell permeability. To examine whether mycolic acid synthesis is altered with DnaK depletion, we analyzed total mycolic acid methyl esters and fatty acid methyl esters made in DnaK replete and depleted cultures. We observed no difference in the amount of total mycolic acid methyl esters or fatty acid methyl esters synthesized within 1 hour in between DnaK replete or depleted cells (Figure S6). Taken together with the FM 4-64 staining and MalF-mCerulean localization, these results indicate that loss of DnaK affects membrane structure and permeability.

### DnaK is required for native firefly luciferase folding in *M. smegmatis*

**Figure 2:**
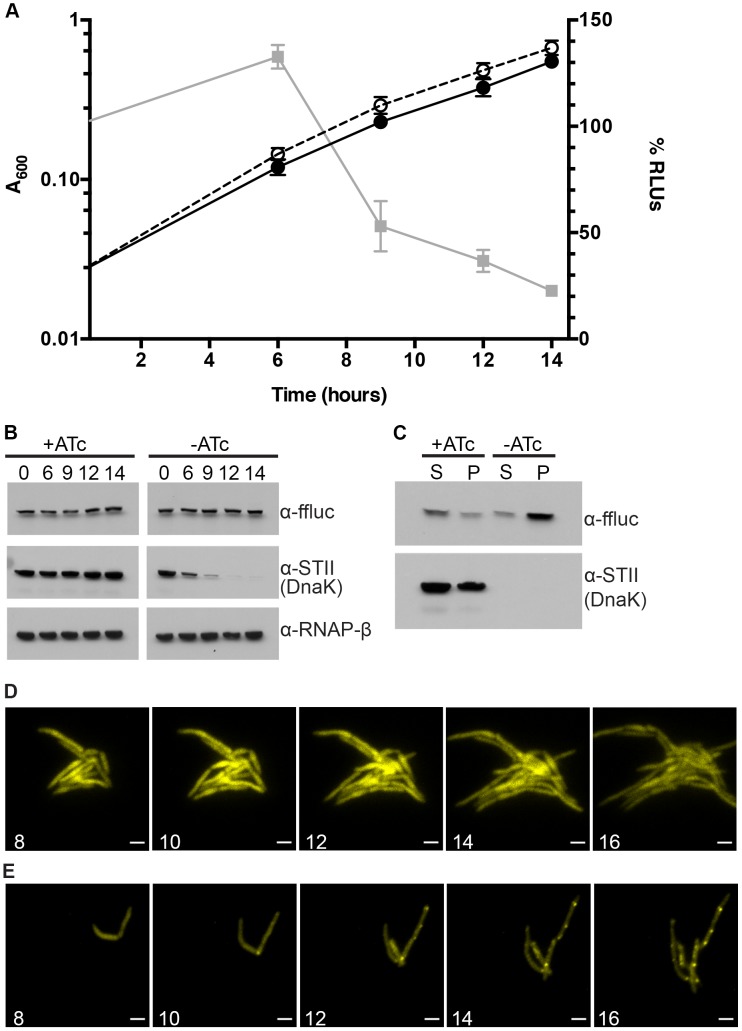
Ms DnaK is required for nascent Firefly luciferase folding. (A) A_600_ and relative Luciferase activity of a Tet-DnaK strain constitutively expressing firefly luciferase (MGM6006). A_600_ (plotted on left Y axis) for +DnaK indicated by closed circles/solid line and for −DnaK as open circles/dashed line. %RLUs (plotted on right Y axis), calculated as Counts per second (CPS) of −DnaK cultures divided by CPS of +DnaK cultures multiplied by 100, indicated by grey squares/solid line. Time indicated on X axis in hours. Each point is the mean of 3 independent cultures. Error bars indicate standard deviation of replicates. (B) Luciferase and Dnak-STII protein levels during DnaK depletion. Immunoblot of lysates prepared from one representative culture of Tet-DnaK Luciferase strain (MGM6006) shown in A. Top panel is probed for firefly luciferase, middle for StrepTagII, bottom for RNAPβ as a loading control. (C) Solubility of luciferase from DnaK depleted cultures. Soluble and pellet protein fractions of lysates prepared from DnaK replete (+ATc) and DnaK depleted (−ATc for 15 hours). Top panel is probed for firefly luciferase, bottom for StrepTagII. (D) Luciferase-mCitrine is diffuse in DnaK replete cells. Time-lapse microscopy of a Tet-DnaK strain constitutively expressing firefly luciferase fused to mCitrine (MGM6010) grown in the presence of ATc. (E) Luciferase-mCitrine forms foci in DnaK depleted cells. Time-lapse microscopy of a Tet-DnaK strain constitutively expressing firefly luciferase fused to mCitrine (MGM6010) grown in the absence of ATc. For (D) and (E), YFP images shown. White numbers indicate time in hours with respect to presence or absence of ATc and white bars indicate 2 µm. Exposure times were YFP 250 ms.

The requirement for DnaK to sustain mycobacterial growth and membrane integrity suggested that DnaK may have a critical role in the absence of exogenous proteotoxic stress. To test the function of DnaK in native protein folding, we expressed firefly luciferase, which has been used as a model protein to study the activity of DnaK in *E. coli* cells after heat shock [33], [34], in our *M. smegmatis dnaK* depletion strain. In cells depleted of DnaK, we observed a rapid loss of luciferase activity (Figure 2A), which occurred prior to growth arrest but coincident with loss of DnaK-STII protein (Figure 2A and B). 14 hours after withdrawal of ATc, >80% of luciferase activity was lost. Although luciferase activity dropped in DnaK depleted cultures, luciferase protein levels remained stable (Figure 2B, Top panel), indicating that the loss of activity was not due to a change in steady state protein levels. By utilizing centrifugation to separate insoluble aggregate proteins, as previously described for *E. coli* [35], we observed that upon DnaK depletion, luciferase was depleted from the soluble fraction and accumulated in the pellet fraction (Figure 2C). Taken together, these results indicate that DnaK is required for folding of luciferase in the absence of heat shock, suggesting a nonredunant role in native protein folding.

To visualize the kinetics and localization of protein aggregate formation without DnaK, we generated a strain carrying a Luciferase-mCitrine fusion protein, which allowed us to simultaneously track luciferase activity and localization during DnaK depletion. Upon depletion of DnaK, the kinetics of loss of luciferase activity from the Luciferase-mCitrine fusion protein were similar to that observed with luciferase (Figure 2A). In contrast, mCitrine fluorescence was maintained in DnaK depleted cells, suggesting that whereas luciferase requires DnaK for folding, mCitrine does not [36]–[38]. Live cell time-lapse imaging in DnaK replete and depleted cells showed two patterns of localization. In the presence of DnaK, Luciferase-mCitrine was cytoplasmic and evenly distributed throughout the cell (Figure 2D). However, upon depletion of DnaK, Luciferase-mCitrine formed polar fluorescent foci which eventually coalesced into large cytoplasmic aggregates (Figure 2E). Immunoblots detecting both Luciferase and Luciferase-mCitrine during DnaK depletion did not detect any proteolytic cleavage of the protein (Figure S7), even after hours of DnaK depletion.

In *E. Coli*, DnaK and Trigger Factor cooperate to support the folding of nascent peptides. Their activities are largely redundant such that, at permissive temperatures, loss of either chaperone is tolerated [13], [14]. To examine potential redundancy between mycobacterial TF and DnaK, we deleted MSMEG_4674, the gene encoding TF (Figure S1C). In contrast to DnaK, mycobacterial TF was nonessential. Furthermore, overexpression of Trigger Factor did not rescue the loss of luciferase activity observed in DnaK depleted cultures (MGM6073) or allow for the loss of the *dnaK* gene (MGM6072) (data not shown). Loss of TF did not affect luciferase activity in a wild type background (Figure S8A) or bacterial growth (Figure S8B). DnaK depletion in the absence of Tigger Factor led to a modest acceleration in the kinetics of luciferase activity loss (Figure S8A), and in the time to growth arrest (Figure S8B). These data indicate that Trigger Factor makes a minor contribution to nascent luciferase folding and stability in *M. smegmatis* that is only evident when DnaK is absent, and demonstrate that DnaK is the dominant chaperone for native folding.

### DnaK depletion leads to an increase in endogenous aggregate proteins

**Figure 3:**
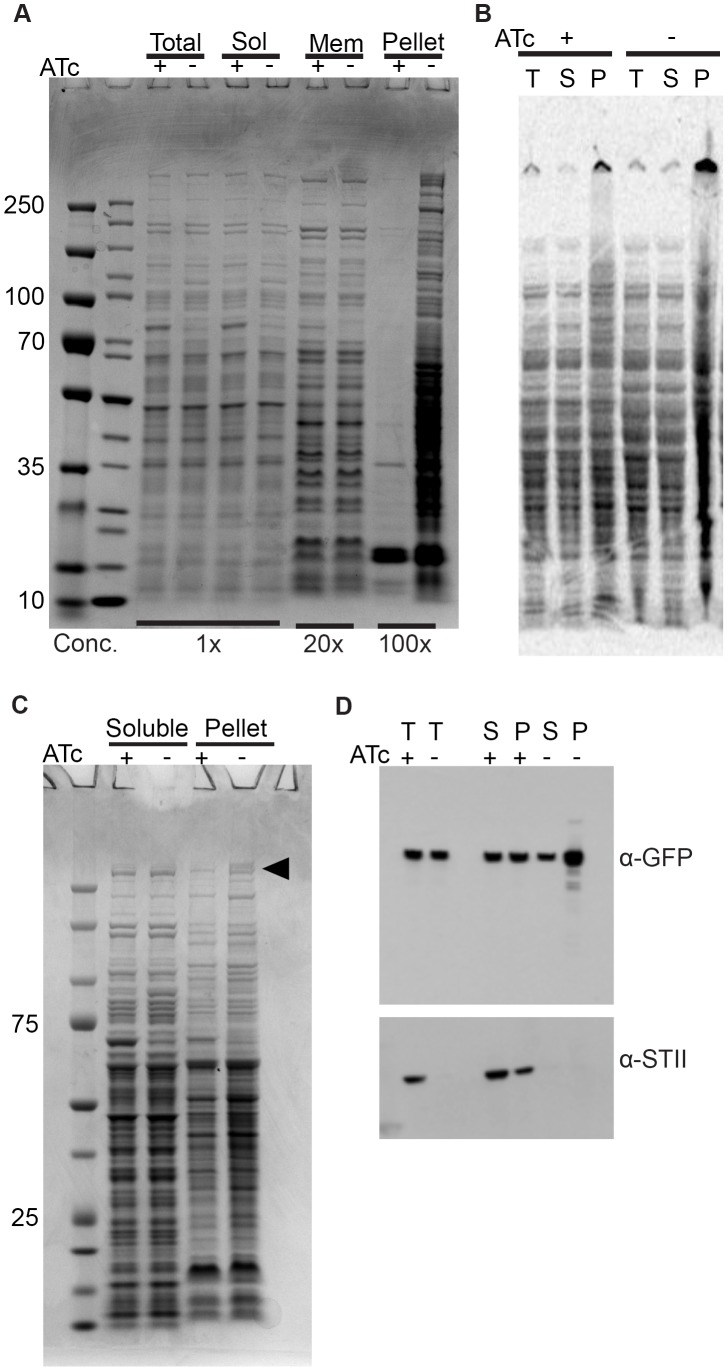
Depletion of Ms DnaK leads to an increase in insoluble protein, including large, multimodular proteins. (A) DnaK depletion leads to an increase in pelleted protein. Coomassie stained SDS-PAGE gel of DnaK replete (+ATc) and DnaK depleted (−ATc) cultures of Tet-DnaK strain (MGM6005) at 16 hours of DnaK depletion. Total, Soluble, Membrane (1% Triton X-100 soluble), and Pelleted (1% Triton X-100 insoluble) fractions shown. (B) DnaK depletion leads to an increase in newly synthesized pellet protein. S^35^-labeled protein fractions from Tet-DnaK strain (MGM6005) cells depleted for DnaK (−ATc) or expressing DnaK (+ATc). Total (T) and soluble (S) fractions at a dilution of 1∶20 as compared to pelleted (P) fraction lanes. (C) DnaK is required for solubility of large multimodular polyketide synthases. Coomassie stained SDS-PAGE gel of DnaK depleted and control cultures of Tet-DnaK strain (MGM6005) at 15 hours post start of depletion. The black arrow indicates high molecular weight proteins that appeared in the pellet fraction in the absence of DnaK. (D) Fatty Acid Synthase I requires DnaK for solubility. Total (T), Soluble (S) and pellet (P) protein fractions of lysates prepared from a Tet-DnaK strain expressing FASI fused at the C-terminus to mCitrine (MGM6014) depleted for 15 hours. Top panel is probed for FASI-mCitrine, bottom for DnaK-STII.

The insolubility of luciferase in DnaK depleted cells suggests a generalized role for DnaK in maintaining native protein folding and solubility. To test this idea, we examined the solubility of endogenous *M. smegmatis* proteins in the absence of DnaK. We depleted DnaK for 16 hours, a time point at which cells are still replicating and maintain full viability (Figure 1A), yet have no detectable DnaK (Figure 1B). We fractionated lysates from DnaK depleted and replete cells and compared fractions by SDS-PAGE. The total, soluble, and membrane (1% Triton X-100 soluble) fractions were similar from depleted and replete cells (Figure 3A). In contrast, the protein content of the pelleted (Triton X-100 insoluble) fraction was substantially increased in lysates from DnaK depleted cells (Figure 3A). To determine whether protein insolubility was the result of nascent protein misfolding versus aggregation of existing protein pools, we performed short term labeling of newly synthesized proteins with ^35^S-Methionine and analyzed the relative incorporation of the label into soluble and insoluble fractions with and without DnaK. In the absence of DnaK, nascent peptides accumulated in the insoluble fraction, accounting for approximately a 45% (±4.8%) increase in insoluble proteins, indicating that DnaK is required for nascent peptide folding (Figure 3B). To exclude an effect of DnaK depletion on translation rates that might account for these findings, we quantitated the rate of nascent chain synthesis using puromycin labeling and detection with an anti-Puromycin antibody [14], [39]. Both DnaK replete and depleted cells produced puromycilated chains at equal rates, as determined by immunoblotting (Figure S9), indicating that DnaK loss does not affect rate of translation.

### DnaK is required for solubility of modular polyketide synthases

Inspection of the proteins that become insoluble without DnaK revealed several high molecular weight proteins in the insoluble fraction (Figure 3C). Mass spectroscopic identification of tryptic peptides derived from these two high molecular weight proteins identified two polyketide synthetases, MSMEG_0408 (type 1 modular polyketide synthase) and MSMEG_0400 (MtbH, peptide synthase) (Figure 3C). Mycobacteria are unusual in that they encode many multimodular polyketide sythases for lipid synthesis, which are very large proteins greater than 300 kDa. The *M. smegmatis* chromosome encodes 8 proteins larger than 2000 amino acids, whereas the *E. coli* K12 chromosome encodes only 1. To extend the finding that DnaK is required for solubility of large multimodular enzymes in mycobacteria, we examined one additional essential large multimodular protein, fatty acid synthase I (FASI, 3089AA). We generated a strain expressing a single full-length copy of FASI fused to mCitrine the C terminus, expressed from its endogenous locus in our DnaK depletion strain background (strain MGM6014). Cell fractionation in DnaK replete or depleted cells revealed that, although some FASI is present in the insoluble fraction with DnaK, FASI accumulated in the insoluble fraction after DnaK depletion (Figure 3D). This demonstrates that FASI, a large, multimodular, essential protein in mycobacteria, requires DnaK for optimal folding and solubility in the absence of proteotoxic stress, suggesting that FASI may be a direct client of DnaK. Taken together our data indicates that DnaK is required for the solubility of at least 3 of the 8 large multimodular proteins in the mycobacterial proteome, all of which are lipid synthases, potentially explaining the disruption of membrane integrity observed in DnaK depleted cells.

### ClpB relocalizes and is up-regulated after DnaK depletion

**Figure 4:**
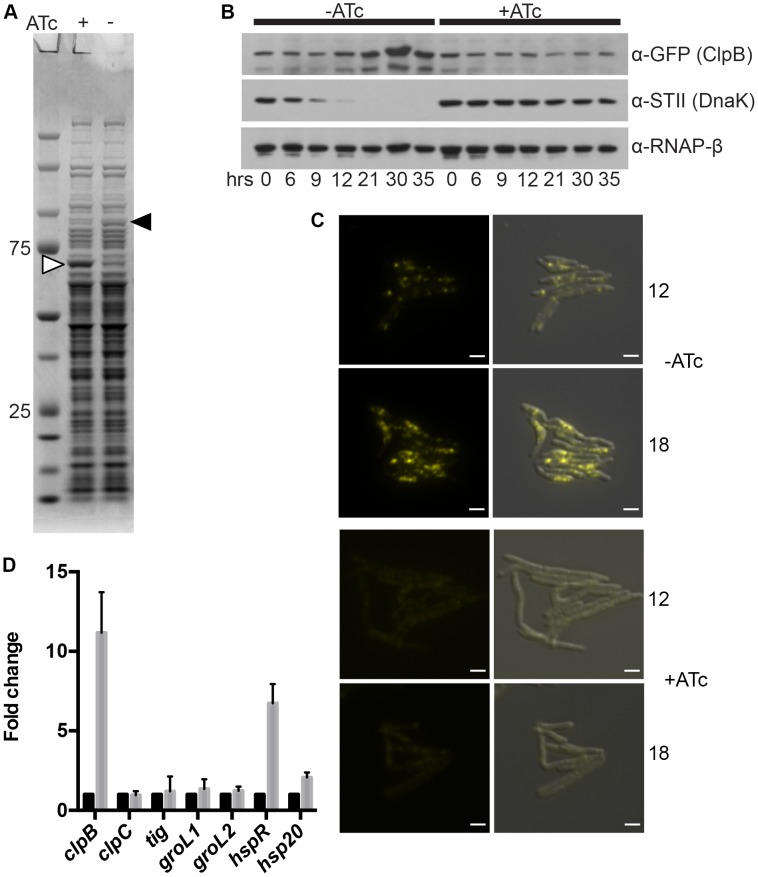
ClpB relocalizes to polar foci and is upregulated after DnaK depletion. (A) DnaK depletion leads to an increase in soluble ClpB. Soluble protein fractions of lysates prepared from a Tet-DnaK strain (MGM6005) replete or depleted for 15 hours. White arrow indicates DnaK-STII, black arrow indicates band identified as ClpB. (B). DnaK depletion leads to an increase in ClpB-mCitrine after 12 hours of depletion. Immunoblot of lysates prepared from DnaK replete or depleted cells of a Tet-DnaK strain expressing ClpB fused at the C-terminus to mCitrine (MGM6008). Top panel is probed for ClpB-mCitrine, middle for StrepTagII, bottom for RNAPβ as a loading control. (C) Images of Tet-DnaK strain expressing ClpB-mCitrine (MGM6008) at 12 and 18 hours into DnaK depletion. Top panels are without DnaK (−ATc), bottom panels are with DnaK (+ATc). White arrow indicates early ClpB-mCitrine foci formed by 12 hours of depletion. Overlay is of YFP and DIC images. Exposure times were YFP 500 ms. White bars indicate 2 µm. (D) *clpB*, but not several other genes encoding chaperones, is upregulated after DnaK depletion. RT-qPCR analysis of the indicated chaperone genes using RNA collected from 3 independent cultures of a Tet-DnaK strain (MGM6005) grown in the presence of absence of ATc for 15 hours. *sigA* was used as a normalization control and all gene pairs are plotted as fold change in comparison to replete cultures.

In addition to the large polyketide synthases that become insoluble in DnaK depleted cells, examination of soluble proteins in SDS-PAGE fractionated lysates from DnaK depleted cells revealed a protein species of approximately 90 kDa that was overrespresented in DnaK depleted cells (Figure 4A, black arrow). Mass spectrometry identified this protein as ClpB. To track both ClpB levels and localization during DnaK depletion, we fused mCitrine to the 3′ end of *clpB* in the DnaK depletion strain (strain MGM6008). ClpB levels were stable in DnaK replete cells (Figure 4B), and during the first 12 hours of DnaK depletion. Beginning at 21 hours after DnaK depletion, ClpB accumulated (Figure 4B). By microscopy, ClpB-mCitrine was expressed at low levels and was near the limit of detection (Figure 4C). At early time points of DnaK depletion, when native folding is lost, but before ClpB protein accumulation by immunoblot (9 to 12 hours), ClpB-mCitrine re-localized to form cytoplasmic foci, suggesting that ClpB relocalizes to protein aggregates that accumulate after loss of DnaK (Figure 4C). In several bacterial species other chaperones have been shown to be upregulated after loss of DnaK to compensate for the defect in chaperone function [10], [40]–[42]. To assess the potential compensatory upregulation of the chaperone network that may respond to DnaK loss in mycobacterial cells, we performed RT-qPCR on RNA collected from control and DnaK depleted cells. We detected upregulation of the mRNAs encoding ClpB, HspR, and Hsp20 (Figure 4D), all most likely the result of destabilization of HspR in the absence of DnaK [17]. No other chaperones or proteases tested were upregulated in the absence of DnaK, indicating that there is a lack of broad compensatory upregulation of alternative chaperone systems to handle the insoluble proteins that accumulate without DnaK function.

### Relocalization of Dnak during proteotoxic stress

**Figure 5:**
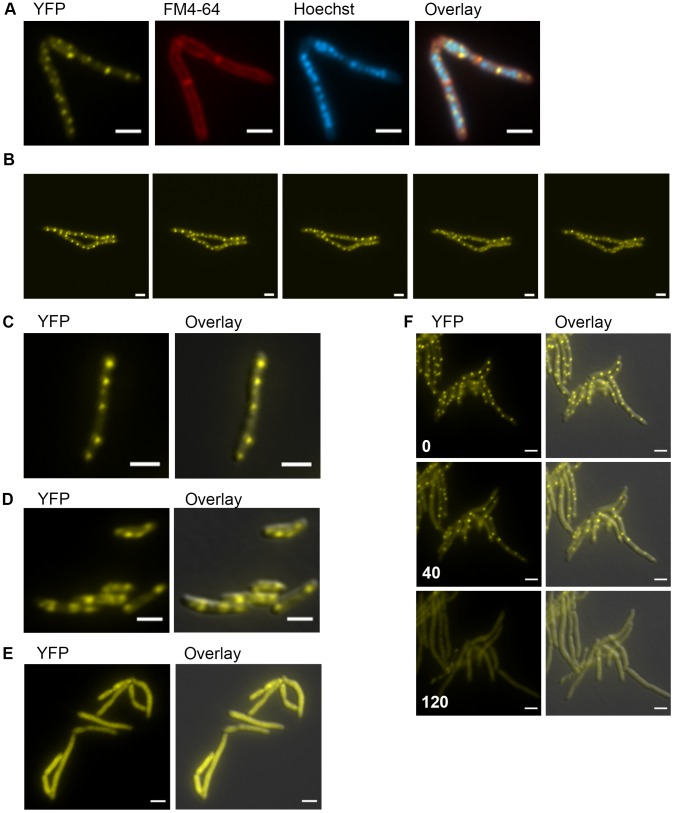
DnaK has growth phase dependent patterns of subcellular localization. (A) DnaK-mCitrine localizes to peripheral foci during growth. Cells expressing DnaK fused at the C-terminus to mCitrine (MGM6003) at A_600_ 0.2 stained with FM 4-64 and Hoechst prior to imaging. (B). DnaK-mCitrine foci are dynamic. Time-lapse microscopy of DnaK-mCitrine expressing strain (MGM6003) at 37°C. YFP images were acquired every 15 minutes as indicated in bottom left corner (Also see Movie S1 and Figure S12). (C). DnaK-mCitrine localizes to peripheral foci in *Mycobacterium bovis* BCG. BCG expressing DnaK fused at the C-terminus to mCitrine (MGM6023) at A_600_ 0.2. Overlay is of YFP and DIC images. (D) DnaK-mCitrine localizes to 1 or 2 large foci in stationary phase cells. Cells of DnaK-mCitrine strain (MGM6003) from late stationary phase culture. Overlay is of YFP and DIC images. (E) Catalytically inactive DnaK fails to localize to peripheral foci. Cells expressing DnaK(K70A)-mCitrine (MGM6024) at A_600_ 0.2. Overlay is of YFP and DIC images. (F) GrpE overexpression disperses DnaK-mCitrine foci. Time-lapse microscopy of Tet-GrpE DnaK-mCitrine expressing strain (MGM6016). Time at lower left of panel indicates minutes after addition of ATc to induce GrpE overexpression. Overlay is of YFP and DIC images. For all images: White bars indicate 2 µm. Exposure times were YFP 250 ms, FM 4-64 500 ms, Hoechst 500 ms.

Based on our findings that mycobacterial DnaK plays a crucial role in native protein folding and in maintaining membrane protein and/or lipid composition, we hypothesized that it might localize in a peri-membrane pattern. In *E. coli*, DnaK localizes in a diffuse cytoplasmic pattern at 37°C and relocalizes to foci at 42°C and above [43], [44]. To assess DnaK localization in *M. smegmatis*, we generated a fully functional DnaK-mCitrine fusion at the native chromosomal locus such that DnaK-mCitrine was expressed as a stable fusion at the estimated full-length size (Figure S10). DnaK-mCitrine appeared to be functional for essential DnaK functions as well as for heat resistance at 53°C (Figure S11) as the DnaK-mCitrine fusion is the only copy of DnaK in the cell. DnaK-mCitrine was visible in multiple membrane peripheral foci distributed along the entire length of the cell during logarithmic growth at 30°C and 37°C (Figure 5A). DnaK foci were dynamic, changing in both number and localization within minutes (Figure 5B, Figure S12A,and Movie S1). The number of foci per micron of cell length varied among cells and within the same cell at different timepoints, however the number of foci per micron had a slightly negative correlation with the length of the cell (Pearson r −0.148, p-value 0.0013)(Figure S12B). So while longer cells had more total foci than shorter cells, they had slightly fewer foci per micron. A similar pattern of DnaK localization was observed in *M. bovis* BCG, indicating that this pattern is conserved across saprophytic and pathogenic mycobacteria (Figure 5C). In stationary phase cells, DnaK-mCitrine dramatically relocalized to form 1 or 2 foci in cells (Figure 5D). This pattern of localization suggested that DnaK re-localized to aggregates formed during stationary phase. To test the role of DnaK function in this localization pattern, we generated an ATPase mutant of DnaK, K70A. Ms DnaK(K70A)-mCitrine failed to complement the DnaK deletion strain and also did not form foci in log or stationary phases (Figure 5E) despite expression as a stable full-length protein at similar levels as wildtype (Figure S10).

Several components of the DnaK chaperone system have been shown to alter the oligomeric state of DnaK in *E. coli* including levels of ATP and GrpE, a DnaK co-factor, [45]. Elevated levels of GrpE inhibit DnaK chaperone activity [46]. We tested the effect of GrpE overexpression on DnaK function and localization. Overexpression of GrpE altered the localization pattern of DnaK such that the dynamic, peripheral foci were lost (Figure 5F and Movie S2). The dispersal of DnaK by GrpE was not due to an effect on DnaK protein levels as the abundance of the DnaK-mCitrine fusion appeared unchanged by immunoblot (Figure S13A). Although prolonged overexpression of GrpE inhibited growth and inhibited luciferase activity, DnaK-mCitrine failed to localize to foci (Figure S13B), indicating that overexpression of GrpE inhibits both the log phase and stationary phase functions of DnaK.

### Relocalization of DnaK-mCitrine and ClpB-mCitrine to protein aggregates

**Figure 6:**
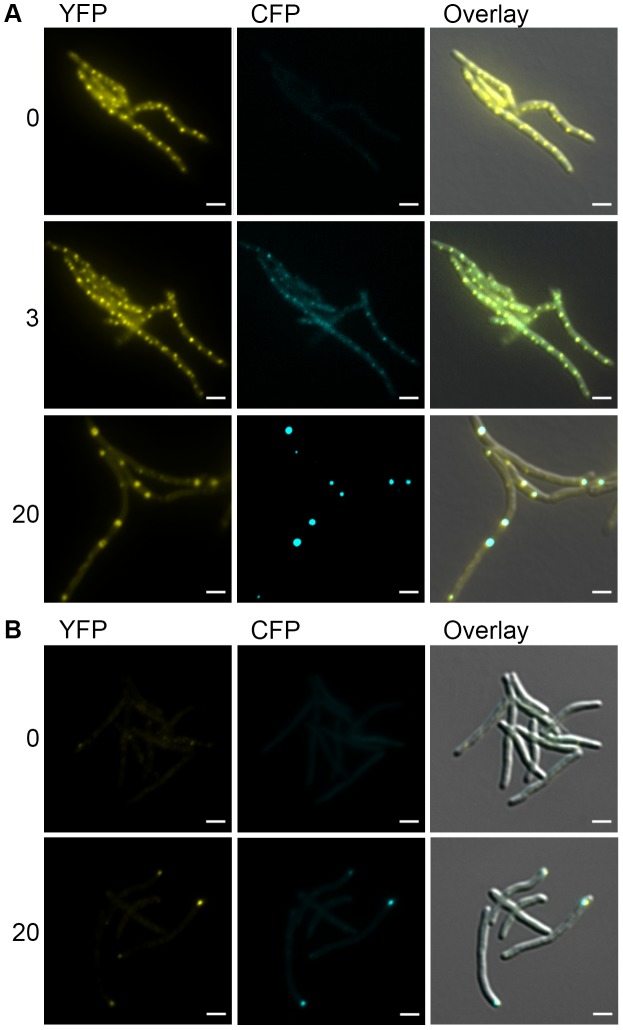
DnaK-mCitrine and ClpB-mCitrine co-localize with protein aggregates. (A) DnaK-mCitrine co-localizes with mCerulean-ELK16. Time-lapse microscopy of DnaK-mCitrine Tet-mCerulean-ELK16 (MGM6011). Time shown after induction of mCerulean-ELK16 in hours to right of panels. Cells shown at 0 and 3 hours are same. Different cells from same experiment shown at 20 hours. (B) ClpB-mCitrine co-localizes with mCerulean-ELK16. Microscopy of ClpB-mCitrine Tet-mCerulean-ELK16 expression strain (MGM6012). Time shown after induction of mCerulean-ELK16 in hours to right of panels. For all images: White bars indicate 2 µm. Exposure times were YFP 250 ms and CFP 500 ms.

The focal relocalization of DnaK in stationary phase cells suggested that DnaK may relocalize to protein aggregates. To test this hypothesis, we induced the formation of protein aggregates by expressing the aggregating protein sequence ELK16 fused to mCerulean and confirmed that mCerulean-ELK16 accumulated in the insoluble fraction (data not shown). We observed mCerulean aggregates accumulating after the induction of mCerulean-ELK16 expression (Figure 6A). With low levels of mCerulean-ELK16 (3 hours after addition of inducer), aggregates colocalized with DnaK in the peripheral foci characteristic of the pattern of DnaK during log phase growth (Figure 6A, 3 hour panel). However, with accumulation of larger mCerulean-ELK16 aggregates, we observed relocalization of DnaK to these larger central aggregates (Figure 6A, 20 hour panel and Movie S3), a pattern that resembles that of DnaK in stationary phase cells. When we observed mCerulean-ELK16 aggregates in a strain expressing ClpB-mCitrine, we observed colocalization of ClpB to the cytoplasmic, but not peripheral, aggregates at late time points (Figure 6B, 20 hour panel). Taken together, these data indicate that DnaK has two modes of chaperone function, one in native protein folding in which it is localized in mobile peripheral foci, and one in aggregate processing in which DnaK relocalizes to central immobile foci of protein aggregates, which also contain ClpB.

### Mycobacterial cells tolerate protein aggregates during cell growth

**Figure 7:**
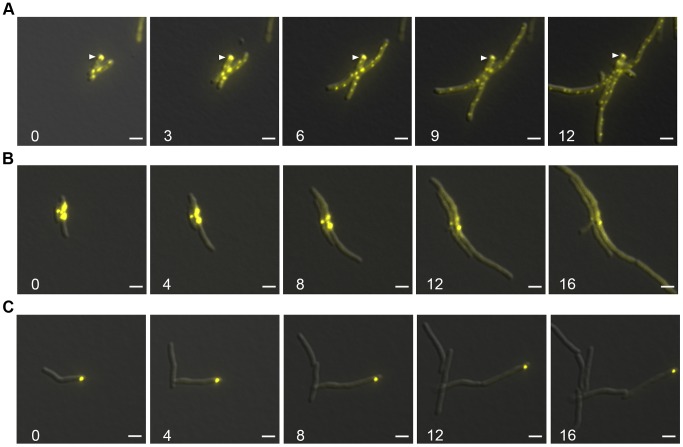
Protein aggregates persist during outgrowth of cells and are partitioned away from growing cells. (A) Large static foci of DnaK-mCitrine persist during outgrowth of cells from stationary phase. Time-lapse microscopy of the DnaK-mCitrine strain (MGM6003). Time in white indicates hours after start of outgrowth. White arrow indicates large static foci that persists for over 12 hours. (B) Luciferase-mCitrine aggregates persist after growth is restored by DnaK expression. Outgrowth Tet-DnaK Luciferase-mCitrine strain (MGM6010) in the presence of ATc after DnaK depletion-induced bacteriostasis. Numbers indicate the time in hours after start of outgrowth. (C) Citrine-ELK16 aggregates persist during outgrowth. Time-lapse microscopy of aggregates during outgrowth after withdrawl of the ATc inducer of Tet-mCitrine-ELK16 expressing strain (MGM6022). For all images: Overlay of YFP and DIC images shown. White bars indicate 2 µm. Exposure times were YFP 250 ms.

We observed that DnaK relocalized to aggregate proteins and that DnaK-mCitrine relocalized to similar patterns during late stationary phase. We next asked whether this aggregate relocalization was reversible by observing the pattern of DnaK localization during outgrowth from stationary phase. We observed that DnaK foci were largely immobile and persisted through several rounds of outgrowth (Figure 7A, white arrow and Movie S4). By 3 hours, larger foci were still visible, but peripheral dynamic foci had reformed. By 12 hours, the original aggregate containing cell still had some immobile DnaK-mCitrine, but all daughter cells contained only dynamic peripheral foci (Figure 7A). This suggested that protein aggregates formed during stationary phase are not dissolved prior to re-growth, but rather are tolerated by the mycobacterial cell for several rounds of division. This experiment also reveals that the two modes of DnaK function can coexist in the cell with DnaK dynamically shuttling between a function in aggregate processing and native folding.

We utilized live cell time-lapse microscopy to directly observe the fate of protein aggregates during cell outgrowth. We first formed luciferase protein aggregates by transiently depleting DnaK, followed by outgrowth with DnaK reexpression. Luciferase-mCitrine aggregates persisted through several cycles of cell division, but cell growth initiated rapidly from the cell pole opposite the aggregate, eventually producing aggregate free cells. (Figure 7B and Movie S5). mCitrine-ELK16 aggregates behaved similarly: aggregates of mCitrine-ELK16 were very stable and persisted in cells after several rounds of division (Figure 7C) with a similar pattern of growth away from the aggregate, eventually forming aggregate free cells, despite persistence of the aggregate in the original cell. Thus, in three different models of protein aggregate formation (stationary phase, DnaK depletion, and heterologous protein expression) protein aggregates are stable during outgrowth and dissolution of aggregates is not required for reinitiation of cell growth. Aggregates were eventually lost in the population by dilution as they remained in the original parent cells but were not divided amongst daughters, suggesting that their loss was passive rather than by an active mechanism such as proteolysis.

## Discussion

We have characterized the function of DnaK in mycobacteria and find that its cellular function differs substantially from what is known about other bacterial HSP70 chaperone systems. Our findings indicate that Mycobacterial DnaK is a central hub of the mycobacterial chaperone network with distinct nonredundant functions in both native and stress induced protein folding. These two states of DnaK function are accompanied by rapid shuttling of DnaK between two cytologic states of protein localization, one that reflects its native folding function and one at cytoplasmic aggregates.

### A nonredundant role for mycobacterial DnaK in native protein folding

Our results indicate that mycobacterial DnaK is the dominant chaperone responsible for folding of native peptides in the absence of exogenous stress such as heat shock. This native folding function is evident both with model protein substrates (luciferase) and endogenous mycobacterial proteins. Trigger Factor in mycobacteria is nonessential and cannot compensate for DnaK loss, even when overexpressed. This contrasts with the function of *E. coli*, in which TF and DnaK have redundant functions in native protein folding and are essential in combination [13], [14].

Although loss of DnaK is accompanied by broad loss of protein solubility and formation of cytoplasmic protein aggregates, we also identified large multimodular lipid synthases as a specific class of proteins that require DnaK for solubility. We show that 3 of the 8 proteins greater than 2000 amino acids in the *M. smegmatis* proteome become insoluble in the absence of DnaK. One of these proteins, fatty acid synthase I (FASI), is a eukaryotic type FAS protein that is not found in bacterial taxa except for the Mycolic acid producing *Actinomycetales* [47] and is essential for viability [48]. The abundance of very large lipid synthases in *Actinomycetales* may mandate distinct chaperone functions to assure proper folding of these large multidomain proteins, as has been shown for eukaryotic proteomes that are enriched for multidomain large proteins in comparison to *E. coli* [49], [50]. Aggregation of large multidomain proteins is a feature of the proteostasis collapse that accompanies combined deletion of DnaK and TF in *E. coli* [7]. This requirement for DnaK in maintaining the solubility of large multimodular lipid synthases is consistent with our finding that the major morphologic and functional perturbation of mycobacterial cells upon growth arrest during DnaK depletion is perturbed membrane structure, rather than the filamentation phenotype seen in *Caulobacter crescentus* [51] and *E. coli* lacking DnaK [6], [52]. This requirement for DnaK to maintain the solubility of the lipid biosynthetic machinery also fits with prior literature demonstrating that GroEL1 in mycobacteria is a specialized chaperone of the FASII enzymes that elongate FASI fatty acid products to Mycolic acids [53], indicating that mycobacteria use a series of chaperones to maintain a functional lipid biosynthetic machinery.

The essential function of DnaK in mycobacteria is also apparently distinct from the mechanism of essentiality recently reported in *C. crescentus*. Protein aggregation that accompanies loss of DnaK activates degradation of DnaA through the Lon protease with consequent cell cycle arrest and filamentation [51]. Overexpression of DnaA restores cell growth in the DnaK mutant strain, indicating that this is the sole determinant of growth arrest in DnaK depleted cells. In our experiments, DnaK depletion in mycobacteria is not accompanied by filamentation, although DnaA depletion in mycobacteria does cause a filamentation phenotype [54]. It is also unlikely that the essentiality of DnaK is due to constitutive activation of the heat shock regulon through loss of HspR repression as previous work in mycobacteria has shown that *hspR* is not essential for growth [55].

### Mycobacterial DnaK in stress induced protein aggregate processing

In addition to its role in native protein folding in unstressed cells, our data also indicate that mycobacterial DnaK also has a second function as a chaperone during states of protein aggregation, akin to the canonical role documented for DnaK in other bacteria. We observed rapid relocalization of DnaK from the dynamic mobile structures characteristic of rapid cell growth to focal protein aggregates that form during stationary phase or by expression of aggregating proteins. These foci remained fixed in both number and intensity and also contain ClpB, suggesting that DnaK and ClpB cooperate in protein aggregate processing, as has been shown in *E. coli* [56].

Once formed, we find that DnaK/ClpB containing protein aggregates are quite stable upon resumption of cell growth and that their dissolution or degradation was not necessary to restart growth after DnaK depletion-mediated growth arrest. This observation suggests that protein aggregates per se are well tolerated by the mycobacterial cell and that cytoplasmic aggregates are not per se toxic if the DnaK system is operative. In this regard, it is relevant that recent work has shown that ClpP1/P2 are essential in mycobacteria [57], [58] and form a mixed heterodimer that constitutes the function ClpP protease [59]. Loss of ClpP leaves cells susceptible to proteotoxic stress and the accumulation of misfolded proteins leading to cell death. Cells depleted for ClpP function have increased susceptibility to streptomycin, an antibiotic that caused mistranslation [58]. The findings presented here identify a second crucial susceptibility point in the mycobacterial chaperone/protein quality control network that could be targeted for antimicrobial development. The nonredunant essential role of Mycobacterial DnaK suggests that small molecule inhibition of this chaperone would be lethal for mycobacteria while sensitizing them to proteotoxic stress induced by the host. Inhibition of chaperone function is an emerging therapeutic strategy in malignant cells that depend on chaperone function and could by similarly targeted as a mechanism to sensitize pathogens to the proteotoxic stress inflicted by the host during infection.

## Materials and Methods

### Bacterial and DNA manipulations

Standard procedures were used to manipulate recombinant DNA and to transform *E. coli*. *M. smegmatis* strains were derivatives of *mc^2^155* [60]. Gene deletions were made by homologous recombination and double negative selection [61]. All strains used in this study are listed in Table S1. Plasmids including relevant features, and primers are listed in Table S2 and S3. *M. smegmatis* was transformed by electroporation (2500 V, 2.5 µF, 1000Ω). All *M. smegmatis* strains were cultured in LB with 0.5% glycerol, 0.5% dextrose (LBsmeg) or 7H9 media for labeling experiments. 0.05% Tween_80_ was added to all liquid media. Heat sensitivity was assayed by incubating cultures at OD_600_ 0.4 at 53°C. Aliquots were taken at indicated time points and serial dilutions were plated on selective media containing ATc. Antibiotic concentrations used for selection of *M. smegmatis* strains were as follows: kanamycin 20 µg/ml, hygromycin 50 µg/ml, streptomycin 20 µg/ml, zeocin 12.5 µg/ml.

### Immunoblotting

For protein and epitope tag detection, the following antibodies were used: StrepTagII (Genescript, Rabbit Anti-NWSHPQFEK polyclonal antibody, 0.5 mg/ml, 1∶40, 000), YFP (Rockland Immunochemicals, Rabbit Anti-GFP polyclonal antibody, 1 mg/ml, 1∶20,000), Luciferase (Millipore, Goat Anti-Luciferase (Firefly) polyclonal antibody, 10 mg/ml 1∶20,000), Puromycin (KeraFast, Mouse anti-Puromycin (3RH11) monoclonal antibody, 1 mg/ml, 1∶2,000), and RNAP-β (Neoclone, 8RB13 Mouse Anti-E. coli RNAPβ monoclonal, 1∶20,000).

### DnaK depletion using ATc withdrawal

Cultures were grown in the presence of 25 ng/ml anhydrotetracycline (ATc) to an OD_600_ of 0.4. Cultures were washed in equal volume of LBsmeg without ATc, then diluted back to indicated OD_600_. This dilution culture was then split and 25 ng/ml ATc was added to one culture, the other was grown in the absence of ATc for depletion. All depletions were carried out in the absence of antibiotic selection.

### Ethidium Bromide uptake assays

A modified previously described method for detecting ethidium bromide uptake was used [31], [32], [62]. Depleted and replete cultures were collected 18 hours after ATc withdrawal, washed in an equal volume of PBS with 0.05% Tween_80_, then resuspended at an OD_600_ 0.4 in PBS with 0.05% Tween_80_ and 0.5% glucose. 95 µl of culture was added to 0.2 ml pcr tubes. For efflux inhibition a final concentration of 5 µM CCCP was added just prior to the start of the assay. The assay was initiated by addition of ethidium bromide (0.25 µg/ml final) in PBS with 0.05% Tween_80_ and 0.5% glucose. Tubes were incubated at 37°C and fluorescence was determined every minute for 60 minutes using an Opticon2 instrument (MJ Research). Experiments were preformed twice in triplicate.

### Mycolate analysis

DnaK depletion was carried out as described above for 16 hours. For labeling, 20 µl of 1-^14^C-Acetic acid, sodium salt (Perkin Elmer, 55.2 mCi/mol) was added to 20 ml of culture incubated at 37°C for 1 hour. Cells were harvested by centrifugation and washed once with 10 ml water. A final suspension of washed cells in 3 ml water was then added to 3 ml 40% tetrabutylammonium hydroxide and incubated at 100°C for 4 hours. An equal volume of dichloromethane was added then 300 µl of methyl iodide and the tubes were then rotated for 1 hour at room temperature. After the liquid layers were allowed to separate the bottom layer was removed and dried overnight. To the dried material, 2 ml ethyl ether was added and the supernatant was moved to a new tube and dried. 100 ul of ethyl ether was added just prior to spotting 5 ul samples on an HPTLC plate (Analtech, 58077). The plate was developed for 4 cycles in a 95∶5 mixture of Hexanes∶ethyl acetate. Plate imaging was preformed using a Phosphor storage cassette and Typhoon Trio (pixel size 200 microns at best sensitivity).

### Luciferase assays

Culture volumes were normalized by OD_600_. A final concentration 5 mM D-luciferin (Gold Biotechnology) was added to each well and Counts per second (CPS) was counted using a Victor2 microplate reader (Perkin Elmer) set to read each well in triplicates, the mean of which was calculated to yield a CPS value for each well. For DnaK depletions %RLUs was calculated by the following equation on biologic triplicate cultures: 100* (CPS_depletion culture_/CPS_control culture_).

### Protein fractionation

#### 3 Step Fractionation (used in Figure 3A)

DnaK depletion was carried out as described above and 50 ml of each culture cooled on ice for 5 min. Cell pellets were collected by centrifugation at 3700 g for 15 minutes at 4°C. Cells were washed with 10 ml buffer A (50 mM Tris, 100 mM NaCl, 10% glycerol, 2 mM EDTA, pH 8) and collected by centrifugation at 3700 g for 15 minutes at 4°C. Glass beads and 1 ml buffer A were added to washed cell pellets and cells were lysed using a Fastprep120 for 3 rounds at 5.0 m/sec for 25 seconds each round with cooling between rounds for 5 minutes on ice and subjected to centrifugation at 3000 g for 5 minutes at 4°C to remove beads and unlysed cells. Supernatants were collected and subjected to a second round of low speed centrifugation at 5000 g for 10 minutes at 4°C. Supernatants from this centrifugation were collected and labeled as “total” and brought up to a volume of 10 ml using buffer A. This total fraction was then centrifuged at 200,000 g for 2 hours at 4°C. Supernatants from this centrifugation were collected as the “soluble” fraction. Pellets were resuspended in 0.5 ml buffer A plus 1% Triton-X 100 by vortexing and allowed to solubilize for 1 hour at 4°C with gentle shaking. This suspension was then centrifuged at 21,130 g for 2 hours at 4°C. The supernatant of this centrifugation was collected as the “membrane” fraction. Pellets were resuspended in 0.1 ml buffer A plus 1% SDS by vortexing and sonication and labeled the “pelleted” fraction.

#### 2 Step Fractionation (used in protein fractionation experiments except for Figure 3A)

DnaK depletion was carried out as described above and 50 ml of each culture cooled on ice for 5 min. Cell pellets were collected by centrifugation at 3700 g for 15 minutes at 4°C. Cells were washed with 10 ml buffer A (50 mM Tris, 100 mM NaCl, 10% glycerol, 2 mM EDTA, pH 8) and collected by centrifugation at 3700 g for 15 minutes at 4°C. Glass beads and 1 ml buffer A were added to washed cell pellets and cells were lysed using a Fastprep120 for 3 rounds at 5.0 m/sec for 25 seconds each round with cooling between rounds for 5 minutes on ice. A final concentration of 0.5% Triton X-100 added to disrupted cells and lysates were left to solubilize for 10 minutes on ice then subjected to centrifugation at 3000 g for 5 minutes at 4°C to remove beads and unlysed cells. Supernatants were collected and subjected to a second round of low speed centrifugation at 5000 g for 10 minutes at 4°C. Supernatants from this centrifugation were collected and labeled as “total”. This total fraction was then centrifuged at 21,130 g for 2 hours at 4°C. Supernatants from this centrifugation were collected as the “soluble” fraction. Pellets were resuspended in buffer A plus 0.5% Triton-X 100 by vortexing and sonication and labeled the “pelleted” fraction.

### Puromycin labeling

DnaK depletion was carried out as described above for 16 hours during which the cultures reached an OD_600_ 0.4. Puromycin was added at 50 µg/ml and the culture was incubated at 37°C. At indicated timepoints cells were harvested by centrifugation and immediately stored at −20°C until completion of the timecourse. For chloramphenicol inhibition controls, 10 µg/ml chloramphenicol was added immediately before the addition of puromycin. Experiments were preformed twice in triplicate.

### ^35^S protein labeling

DnaK depletion was carried out in 7H9 media for 15 hours at 37°C until cultures reached OD_600_ of approximately 0.4. Cultures were normalized to OD_600_ 0.4 and 25 mls of culture was used for labeling. To label, 12.5 µl of Trans^35^S-LABEL (MP Biomedicals, 10.4 mCi/ml) was added and cultures were incubated with shaking for 30 minutes at 37°C. After 30 minutes 1 mM methionine was added and cultures were cooled to 4°C prior to harvesting by centrifugation. Collected cultures were fractionated for Soluble/Pelleting protein analysis as described above for the 2 Step Fractionation. After separation by SDS-PAGE, gels were dried and radioactivity quantitated using Phosphor storage cassette and Typhoon Trio (pixel size 200 microns at best sensitivity). ImageJ was used to quantitate the total radioactive signal per lane.

### Microscopy

All images were acquired using a Zeiss Axio Observer Z1 microscope equipped with Definite focus, Stage top incubator (Insert P Lab-Tek S1, TempModule S1), Colibri.2 and Illuminator HXP 120 C light sources, a Hamamatsu ORCA-Flash4.0 CMOS Camera and a Plan-Apochromat 100×/1.4 oil DIC objective. Zeiss Zen software was used for acquisition and image export. The following filter sets and light sources were used for imaging: YFP (46 HE, Colibri2.0 505 LED), CFP (47 HE, HXP 120 C), Hoechst 33342 (49 HE, HXP 120 C), FM 4-64 (20, HXP 120 C). For cell staining 100 µl of culture was used. A final concentration of 1 µg/ml FM 4-64 (Invitrogen) and/or 10 µg/ml Hoechst 33342 (Invitrogen) was added. Cells were pelleted by centrifugation at 5000 g for 1 minute and resuspended in 50 µl of media. For single time point live cell imaging, 7 µl of culture was spotted onto a No. 1.5 coverslip and pressed to a slide. For time-lapse microscopy, cells were added to a 1.5% Low melting point agarose LBsmeg pad with or without 25 ng/ml ATc. For pad preparation, LBsmeg agarose was heated to 65°C and poured into a 17×28 mm geneframe (Thermoscientific, AB-0578) adhered to a 25×75 mm glass slide. A second slide was pressed down on top and the set-up was allowed to cool at room temperature for 10 minutes. The top slide was removed and the pad was cut and removed so that a 3–4 mm strip remained near the center. 2–3 µl of *M. smegmatis* culture was added to the pad and a No. 1.5 24×40 mm coverglass was sealed to the geneframe. Slides were incubated in stage top incubator at 37°C. For Luciferase-mCitrine aggregate imaging, DnaK depletion was carried out for 6 hours prior to transferring DnaK depleted cells to the pad. For Luciferase-mCitrine aggregate outgrowth imaging, DnaK depletion was performed for 24 hours prior to transferring DnaK depleted cells to a pad containing ATc to reinduce DnaK expression.

### RT-qPCR

50 ml of cultures normalized to an OD_600_ of 0.4 were cooled to 4°C and harvested by centrifugation. Pellets were washed in 1 ml 10 mM Tris, pH 8.0 Pellets were then resuspended in 100 µl TE_80_ with 1 mg/ml lysozyme and disrupted by bead beating with a FastPrep120 2 times at 5.0 m/sec for 25 seconds. This lysate was used for RNA purification with a GeneJet RNA purification kit (Thermoscientific) following the manufacturer's protocol. RNA was eluted in 85 µl elution buffer and then treated with DNase I (Thermoscientific) for 30 minutes at 37°C. GeneJet purification columns were used to clean RNA from DNaseI reactions. First strand cDNA synthesis was carried out using Maxima Universal First Strand cDNA synthesis kit (Thermoscientific) with random hexamers and 500 ng RNA. For each RNA sample a no RT control was used to assess DNA contamination. qPCR was performed using DyNamo SYBR green qPCR kit (Thermoscientific) and an Opticon2 instrument (MJ Research). For each gene, normalized cycle threshold, C(t), was calculated using housekeeping gene *sigA*, and relative expression level was calculated using the equation 2^−(C(t) gene – C(t) sigA)^. All RT-qPCR experiments were performed 2 times in triplicate.

### Protein identification by nano-Liquid Chromatography coupled to tandem Mass Spectrometry (LC-MS/MS) analysis

Proteins were resolved using SDS-polyacrylamide gel electrophoresis, followed by staining with Coomassie Blue and excision of the separated protein bands; *In situ* trypsin digestion of polypeptides in each gel slice was performed as described [63]. The tryptic peptides were purified using a 2-µl bed volume of Poros 50 R2 (Applied Biosystems, CA) reversed-phase beads packed in Eppendorf gel-loading tips [64]. The purified peptides were diluted to 0.1% formic acid and then subjected to nano-liquid chromatography coupled to tandem mass spectrometry (nanoLC-MS/MS) analysis as detailed [65].

Initial protein/peptide identifications from the LC-MS/MS data were performed using the Mascot search engine (Matrix Science, version 2.3.02; www.matrixscience.com) with the Eubacteria segment of Uniprot protein database (12,115,765 sequences; European Bioinformatics Institute, Swiss Institute of Bioinformatics and Protein Information Resource). The search parameters were as follows: (i) two missed cleavage tryptic sites were allowed; (ii) precursor ion mass tolerance = 10 ppm; (iii) fragment ion mass tolerance = 0.8 Da; and (iv) variable protein modifications were allowed for methionine oxidation, cysteine acrylamide derivatization and deamidation of asparagines. MudPit scoring was typically applied using significance threshold score p<0.01. Decoy database search was always activated and, in general, for merged LS-MS/MS analysis of a gel lane with p<0.01, false discovery rate averaged around 1%. Scaffold (Proteome Software Inc., Portland, OR), version 4_1_1 was used to further validate and cross-tabulate the tandem mass spectrometry (MS/MS) based peptide and protein identifications. Protein and peptide probability was set at 99% with a minimum peptide requirement of 1.

## Supporting Information

Figure S1 Southern blot confirmation of *dnaK*, *grpE*, and *tig* allelic replacements. Expected band sizes indicated in parentheses. (A) Confirmation of *dnaK* deletion using EcoRV/NsiI double digest of genomic DNA and *dnaK* 5′ flank as probe. Lanes numbered at top of blot. Lane (1) wt (4647 bp), (2) wt with pAJF510 after 5′ integration at the *dnaK* locus (3848 and 5383 bp), (3) intermediate with pAJF223 (*attB::dnaK*) (3848, 5383, and undetermined), (4) and (5) two Suc^R^/2-DOG^R^ strains from counterselection of the strain in lane #3 yielding either the wt *dnaK* locus (lane 4, 4647 bp and undetermined) or Δ*dnaK* (lane 5, MGM6002, 2758 bp and undetermined). (B) Confirmation of *grpE* deletion using NotI/NsiI double digest of genomic DNA and *grpE* 3′ flank as probe. Lane (1) wt (3348 bp) (2) wt with pAJF509 (*attB::grpE*) and pAJF325 after 3′ integration at *grpE* locus (2711 and 8436 bp), (3) and (4) Suc^R^/2-DOG^R^ strain from counterselection of the strain in lane #2 yielding Δ*grpE*, MGM6027 (lane 3, 2074 bp) or wt (lane 4, 3348 bp). (C) Confirmation of pAJF273 integration for *tig* deletion using *tig* 3′ flank and genomic DNA digests with BamHI/ClaI. Lane (1) Ladder, (2) wt (4279 bp), (3) wt with pAJF273 after 5′ integration at *tig* locus (3128 and 5030 bp), (4) wt (4279 bp). (D) Confirmation of *tig* deletion deletion using *tig* 3′ flank and genomic DNA digests with BamHI/BglII. Lane (1) wt (2813 bp), (2) and (3) two Suc^R^/2-DOG^R^ strains from counterselection of the strain in lane #2 yielding Δ*tig*, MGM6028 (lane 2, 1733 bp) or wt (lane 3, 2813 bp),
(TIF)

Figure S2 *M. smegmatis dnaK* and *grpE* are essential for growth. Strains carrying deletions in chromosomal *dnaK* (A) or *grpE* (B) and a copy of *dnaK* or *grpE* at the *attB* phage integration site were subjected to marker exchange with *attB* integrating vectors. (A) Δ*dnaK attB::dnaK strep* (MGM6002) transformed with pMV306kan (empty vector) or pAJF447 (encoding DnaK-TwinStrep). Transformations on kanamycin selective media incubated at 37°C (top) or 30° (bottom). (B) Δ*grpE attB::grpE strep* (MGM6027) transformed with kanamycin resistance encoding vectors pMV306kan (empty vector) or pAJF509 (encoding GrpE). Transformations on kanamycin selective media incubated at 37°C (top) or 30° (bottom).
(TIF)

Figure S3 DnaK depleted cells are heat sensitive. Heat sensitivity of MGM6005 depleted for DnaK for 12 hours (open triangles, dashed line) or not depleted (closed triangles, solid line). CFU/ml is plotted on a logarithmic Y axis and time of incubation at 53°C on the X axis.
(TIF)

Figure S4 DnaK depleted cells show colocalzing alterations in membrane structure and membrane protein localization. Tet-DnaK, MalF-mCerulean expression strain, (MGM6015) depleted of DnaK for 24 hours (top panels) or DnaK replete (bottom panels). Cells were stained with FM 4-64 just prior to imaging. White bars indicate 2 µm. Exposure times were FM 4-64 500 ms and CFP 500 ms.
(TIF)

Figure S5 Loss of DnaK results in increased membrane permeability. Ethidium bromide uptake assay using MGM6005 18 hours after ATc withdrawal (closed triangles) or DnaK replete (closed circles). Efflux inhibitor, CCCP, added just prior to start of assay to depleted cells (open triangles) and replete cells (open circles). Time indicated at bottom as minutes after the addition of ethidium bromide. Y axis is fluorescent units calibrated for ethidium bromide (see materials and methods).
(TIF)

Figure S6 Loss of DnaK does not affect synthesis of fatty acid methyl esters (FAMEs) or mycolic acid methyl esters (MAMEs). ^14^C-labeled lipids from MGM6005 16 hours after ATc withdrawal to deplete DnaK (−), or with DnaK (+). Triplicate cultures are shown.
(TIF)

Figure S7 Luciferase-mCitrine protein remains stable during DnaK depletion and upon restoration of DnaK expression during outgrowth. Lysates from the Tet-DnaK, Luciferase-mCitrine expression strain (MGM6010) prepared during 24 hours of ATc withdrawal and continued through 6 hours of subsequent induction of DnaK with ATc and outgrowth. Luciferase-mCitrine runs as stable protein at estimated full length, 88 kDa, during DnaK depletion and outgrowth. Immunoblots probed for GFP (Luciferase-mCitrine, middle panel), StrepTagII (DnaK-STII, bottom panel) and RNAP-β as loading control (top panel).
(TIF)

Figure S8 Luciferase activity and growth arrest of DnaK depletion in the presence and absence of Trigger Factor. (A) A_600_ and relative Luciferase activity of a Tet-DnaK strain constitutively expressing firefly luciferase, MGM6006 (+TF) and MGM6071 (−TF). A_600_ (plotted on left Y axis) for +DnaK/+TF indicated by closed circles/black line, for −DnaK/+TF as open circles/grey line, for +DnaK/−TF indicated by closed triangles/black line, and for −DnaK/−TF as open triangles/grey line, for +DnaK/−TF indicated by closed triangles/solid line. %RLUs (plotted on right Y axis), calculated as Counts per second (CPS) of –DnaK cultures divided by CPS of +DnaK cultures multiplied by 100, +TF indicated by closed squares/solid line and −TF indicated by open squares/dashed line. Time indicated on X axis in hours. Each point is the mean of 3 independent cultures. Error bars indicate standard deviation of replicates. (B) Growth of DnaK replete and depleted cells in the presence or absence of TF. A_600_ (plotted on left Y axis) for +DnaK/+TF indicated by closed circles/solid line, for −DnaK/+TF as open circles/dashed line, for +DnaK/−TF indicated by closed triangles/solid line, and for −DnaK/−TF as open triangles/dashed line, for +DnaK/−TF indicated by closed triangles/solid line.
(TIF)

Figure S9 DnaK depleted and replete cells have similar translation rates. Puromycin incorporation in Tet-DnaK strain, MGM6005 started 16 hours after ATc withdrawal. Time indicated at bottom as minutes after addition of 50 µg/ml puromycin-HCl. The right panel is chloramphenicol treated controls for translation inhibition. Immunoblots probed for Puromycin (top panel), StrepTagII (DnaK-STII), and RNAP-β as loading control (bottom panel).
(TIF)

Figure S10 DnaK-mCitrine and DnaK(K70A)-mCitrine are expressed as stable proteins at predicted size (100 kDa). Immunoblot of lysates made from DnaK-mCitrine expression strain (MGM6003), lane 1, and DnaK(K70A)-mCitrine (MGM6024), lane 2. Immunoblots probed for GFP (DnaK-mCitrine, bottom panel) and RNAP-β as loading control (top panel).
(TIF)

Figure S11 Heat sensitivity of strains expressing tagged DnaK and ClpB. Heat sensitivity of MGM6005 depleted for DnaK for 12 hours (grey open squares, dashed line) or not depleted (grey closed square, solid line), wildtype (closed circles/solid line), MGM6003 (DnaK-mCitrine, closed triangles/dashed line), and MGM6009 (ClpB-mCitrine, closed diamonds/dashed line). CFU/ml is plotted on the Y axis and time of incubation at 53°C on the X axis.
(TIF)

Figure S12 DnaK foci are dynamic in growing cells and their density does not reflect difference in cell length. The number of DnaK foci were quantitated for 76 individual cells over 6 timepoints from timelapse micrscopy images. (A) Change in foci in each cell of the 76 cells over time. Relative change in number of foci compared to timepoint 1 plotted on the Y axis for each of the 6 timepoints plotted on the X axis. (B) Foci per micron of cell length as plotted against cell length. Each of the 76 cells plotted for all 6 timepoints for a total of 456 points. Foci/µm plotted on the Y axis and length of cell plotted on the X axis. Linear regression calculated is shown with blue line.
(TIF)

Figure S13 GrpE overexpression impairs DnaK-mCitrine chaperone function, but not DnaK-mCitrine protein stability. (A) Immunoblot of lysates from DnaK-mCitrine, Luciferase and Tet-GrpE expression strain (MGM6016) after 0, 1.5, and 3 hours of GrpE induction with ATc. Immunoblots probed for RNAP-β as loading control (top panel), GFP (DnaK-mCitrine, middle panel), and STII (GrpE-STII, bottom panel). (B) Luciferase activity of MGM6016 up to 6 hours after start of GrpE-STII induction with ATc. %RLUs (plotted on Y axis) calculated as (CPS (−ATc)/CPS(+ATc))*100. Time indicated on X axis in hours. Each point is the mean of 3 independent cultures.
(TIF)

Table S1 Bacterial strains used in this study. Listed are the strain name, relevant genotype, source and details of construction for each strain.
(PDF)

Table S2 Plasmids used in this study. Listed are the plasmid name, relevant features, and details of construction for each plasmid used.
(PDF)

Table S3 Oligonucleotides used in this study. Listed are the oligo name, sequence, and (for qPCR oligos) the target gene amplified.
(PDF)

Movie S1 Timelapse microscopy of DnaK-mCitrine expression strain (MGM6003) at 37°C. Images acquired every 20 minutes for a total elapsed time of 10 hours 40 min. YFP and DIC overlay shown at 3 frames per second (fps). YFP exposure time 250 ms.
(AVI)

Movie S2 Timelapse microscopy of DnaK-mCitrine in the Tet-GrpE expression strain (MGM6010). Cells added to LBsmeg agarose pad containing ATc for induction of GrpE overexpression. Images acquired every 20 minutes for a total elapsed time of 16 hours. YFP and DIC overlay shown at 3 fps. YFP exposure time 250 ms.
(AVI)

Movie S3 Timelapse microscopy of DnaK-mCitrine in the Tet-Cerulean-ELK strain (MGM6011). Cells added to LBsmeg agarose pad containing ATc for induction of mCerulean-ELK16 expression. Left panel is Overlay (YFP/CFP/DIC), middle panel is CFP (cerulean-ELK16), and right panel is YFP (DnaK-mCitrine). Images acquired every 20 minutes for a total elapsed time of 10 hours. YFP and DIC overlay shown at 3 fps. Exposure times were YFP 250 ms, CFP 500 ms.
(AVI)

Movie S4 Timelapse microscopy of DnaK-mCitrine expression strain (MGM6003) during outgrowth from stationary phase. Images acquired every 20 minutes. YFP and DIC overlay shown at 3 fps for a total elapsed time of 17 hours. YFP exposure time 250 ms.
(AVI)

Movie S5 Timelapse microscopy of outgrowth of Tet-DnaK Luciferase-mCitrine strain (MGM6010) in the presence of ATc after DnaK depletion-induced bacteriostasis. Images acquired every 20 minutes for a total elapsed time of 15 hours 20 minutes. YFP and DIC overlay shown at 3 fps. YFP exposure time 250 ms.
(AVI)
