# Supplementary figures and images for: An Essential Nonredundant Role for Mycobacterial DnaK in Native Protein Folding
Source: PLoS Genet. 2014 Jul 24;10(7):e1004516. doi: 10.1371/journal.pgen.1004516 (PMC4109909; doi:10.1371/journal.pgen.1004516)

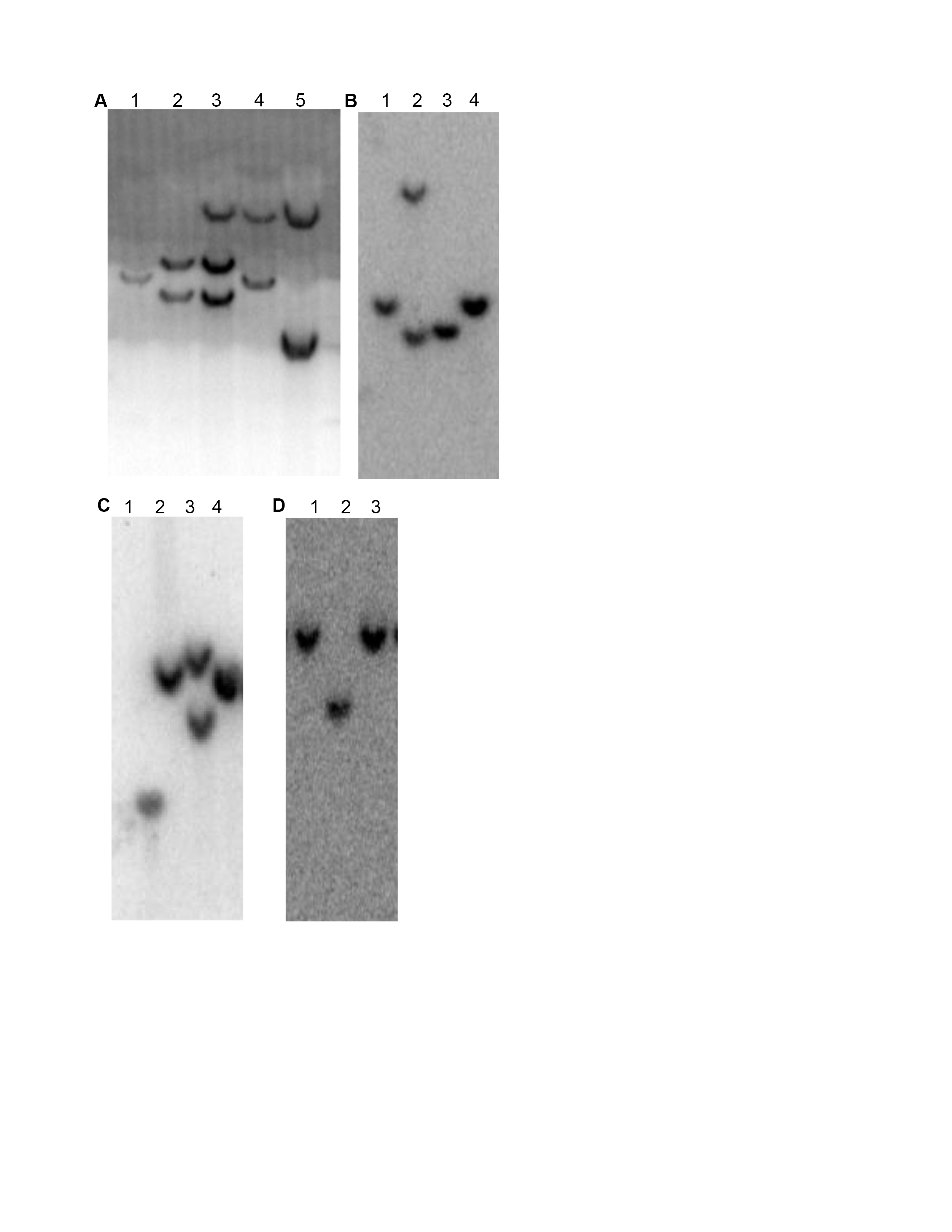

Supplement: Figure S1 — Southern blot confirmation of dnaK, grpE, and tig allelic replacements. Expected band sizes indicated in parentheses. (A) Confirmation of dnaK deletion using EcoRV/NsiI double digest of genomic DNA and dnaK 5′ flank as probe. Lanes numbered at top of blot. Lane (1) wt (4647 bp), (2) wt with pAJF510 after 5′ integration at the dnaK locus (3848 and 5383 bp), (3) intermediate with pAJF223 (attB::dnaK) (3848, 5383, and undetermined), (4) and (5) two SucR/2-DOGR strains from counterselection of the strain in lane #3 yielding either the wt dnaK locus (lane 4, 4647 bp and undetermined) or ΔdnaK (lane 5, MGM6002, 2758 bp and undetermined). (B) Confirmation of grpE deletion using NotI/NsiI double digest of genomic DNA and grpE 3′ flank as probe. Lane (1) wt (3348 bp) (2) wt with pAJF509 (attB::grpE) and pAJF325 after 3′ integration at grpE locus (2711 and 8436 bp), (3) and (4) SucR/2-DOGR strain from counterselection of the strain in lane #2 yielding ΔgrpE, MGM6027 (lane 3, 2074 bp) or wt (lane 4, 3348 bp). (C) Confirmation of pAJF273 integration for tig deletion using tig 3′ flank and genomic DNA digests with BamHI/ClaI. Lane (1) Ladder, (2) wt (4279 bp), (3) wt with pAJF273 after 5′ integration at tig locus (3128 and 5030 bp), (4) wt (4279 bp). (D) Confirmation of tig deletion deletion using tig 3′ flank and genomic DNA digests with BamHI/BglII. Lane (1) wt (2813 bp), (2) and (3) two SucR/2-DOGR strains from counterselection of the strain in lane #2 yielding Δtig, MGM6028 (lane 2, 1733 bp) or wt (lane 3, 2813 bp), (TIF) [file pgen.1004516.s001.tif]

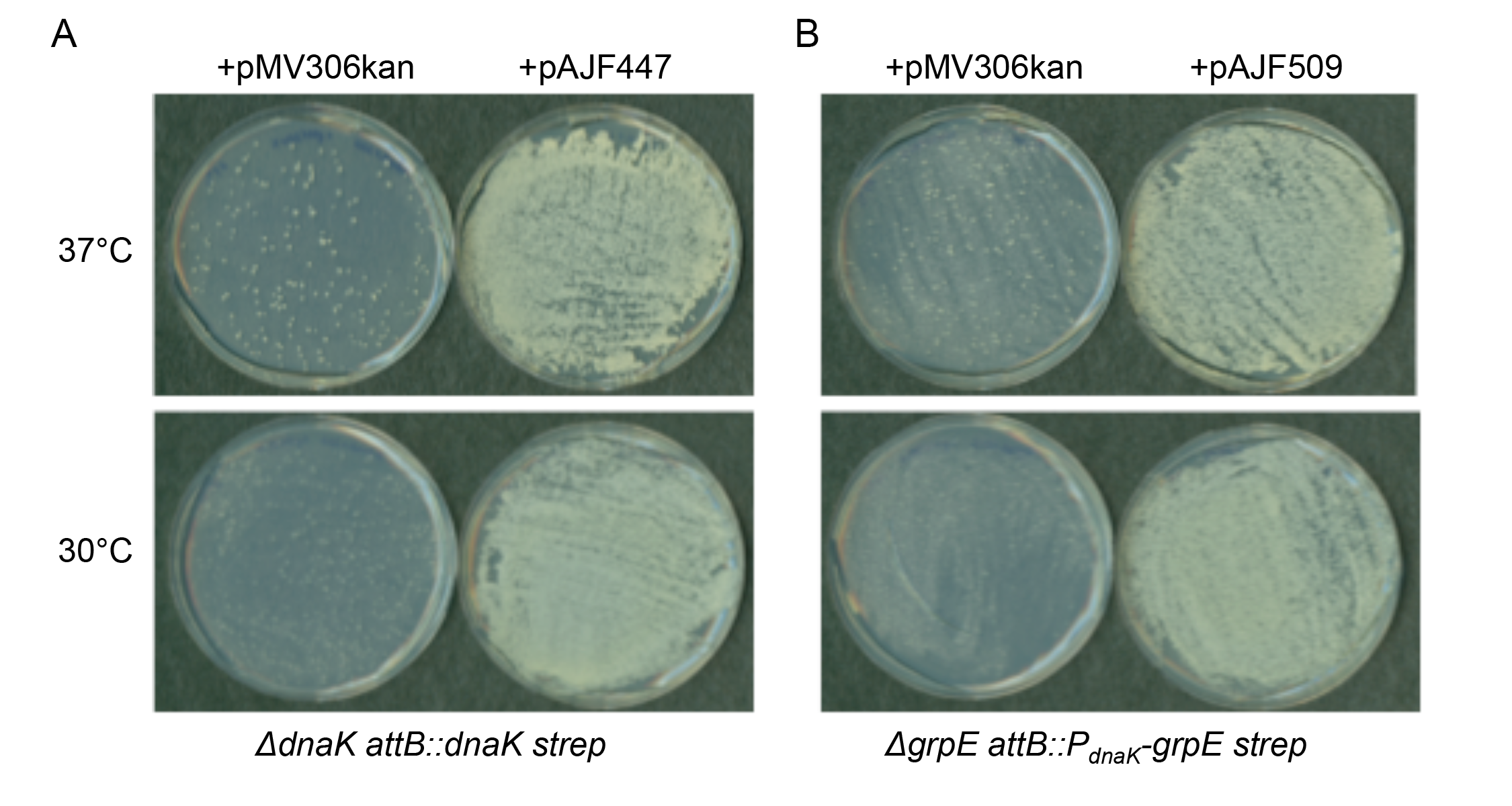

Supplement: Figure S2 — M. smegmatis dnaK and grpE are essential for growth. Strains carrying deletions in chromosomal dnaK (A) or grpE (B) and a copy of dnaK or grpE at the attB phage integration site were subjected to marker exchange with attB integrating vectors. (A) ΔdnaK attB::dnaK strep (MGM6002) transformed with pMV306kan (empty vector) or pAJF447 (encoding DnaK-TwinStrep). Transformations on kanamycin selective media incubated at 37°C (top) or 30° (bottom). (B) ΔgrpE attB::grpE strep (MGM6027) transformed with kanamycin resistance encoding vectors pMV306kan (empty vector) or pAJF509 (encoding GrpE). Transformations on kanamycin selective media incubated at 37°C (top) or 30° (bottom). (TIF) [file pgen.1004516.s002.tif]

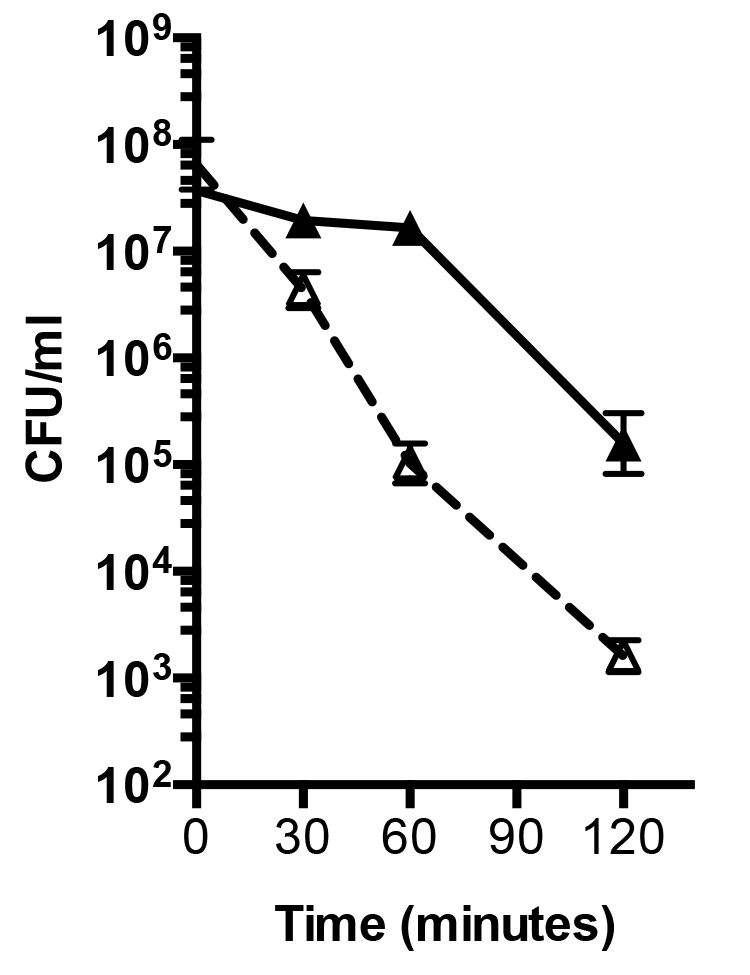

Supplement: Figure S3 — DnaK depleted cells are heat sensitive. Heat sensitivity of MGM6005 depleted for DnaK for 12 hours (open triangles, dashed line) or not depleted (closed triangles, solid line). CFU/ml is plotted on a logarithmic Y axis and time of incubation at 53°C on the X axis. (TIF) [file pgen.1004516.s003.tif]

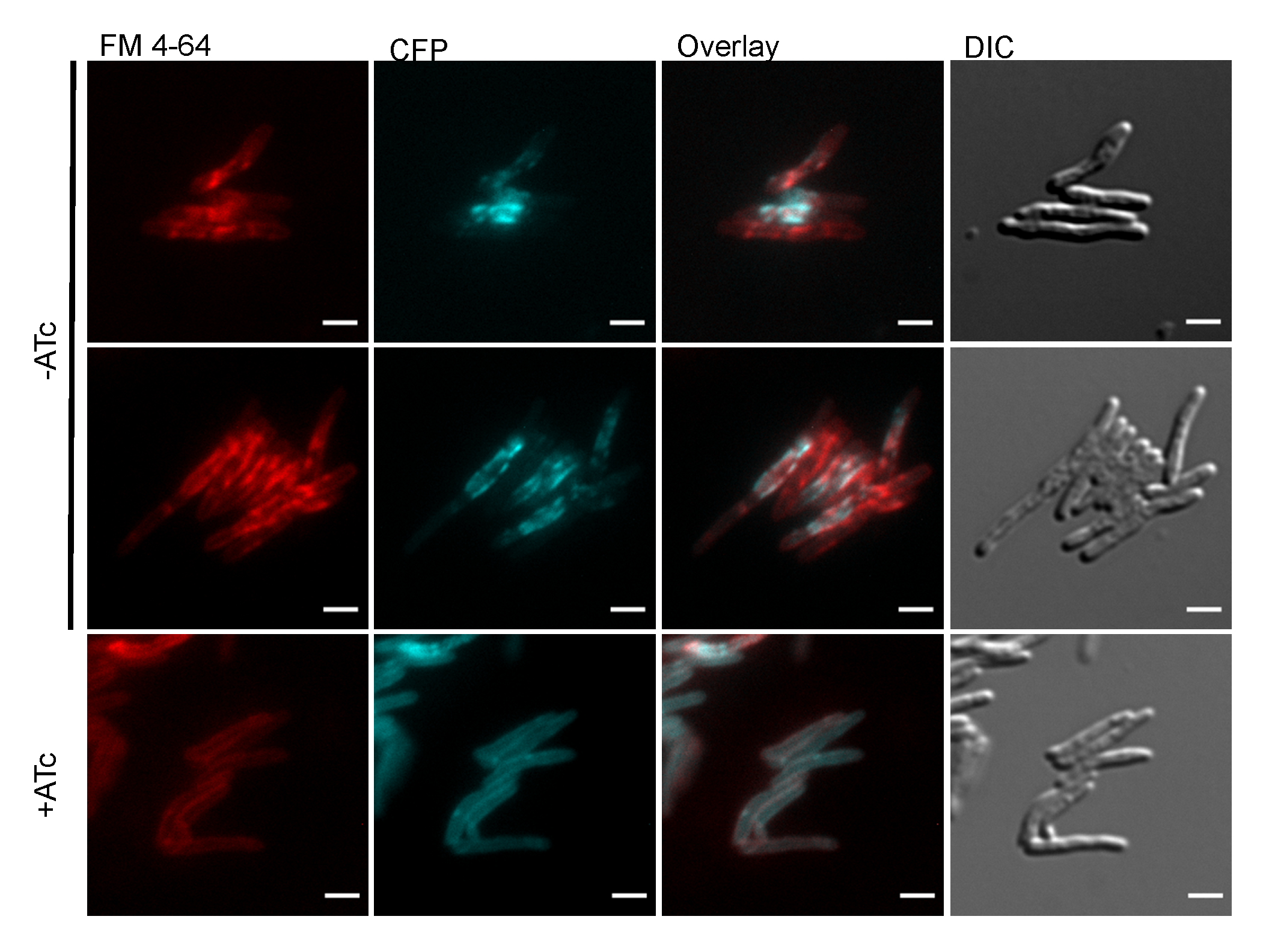

Supplement: Figure S4 — DnaK depleted cells show colocalzing alterations in membrane structure and membrane protein localization. Tet-DnaK, MalF-mCerulean expression strain, (MGM6015) depleted of DnaK for 24 hours (top panels) or DnaK replete (bottom panels). Cells were stained with FM 4-64 just prior to imaging. White bars indicate 2 µm. Exposure times were FM 4-64 500 ms and CFP 500 ms. (TIF) [file pgen.1004516.s004.tif]

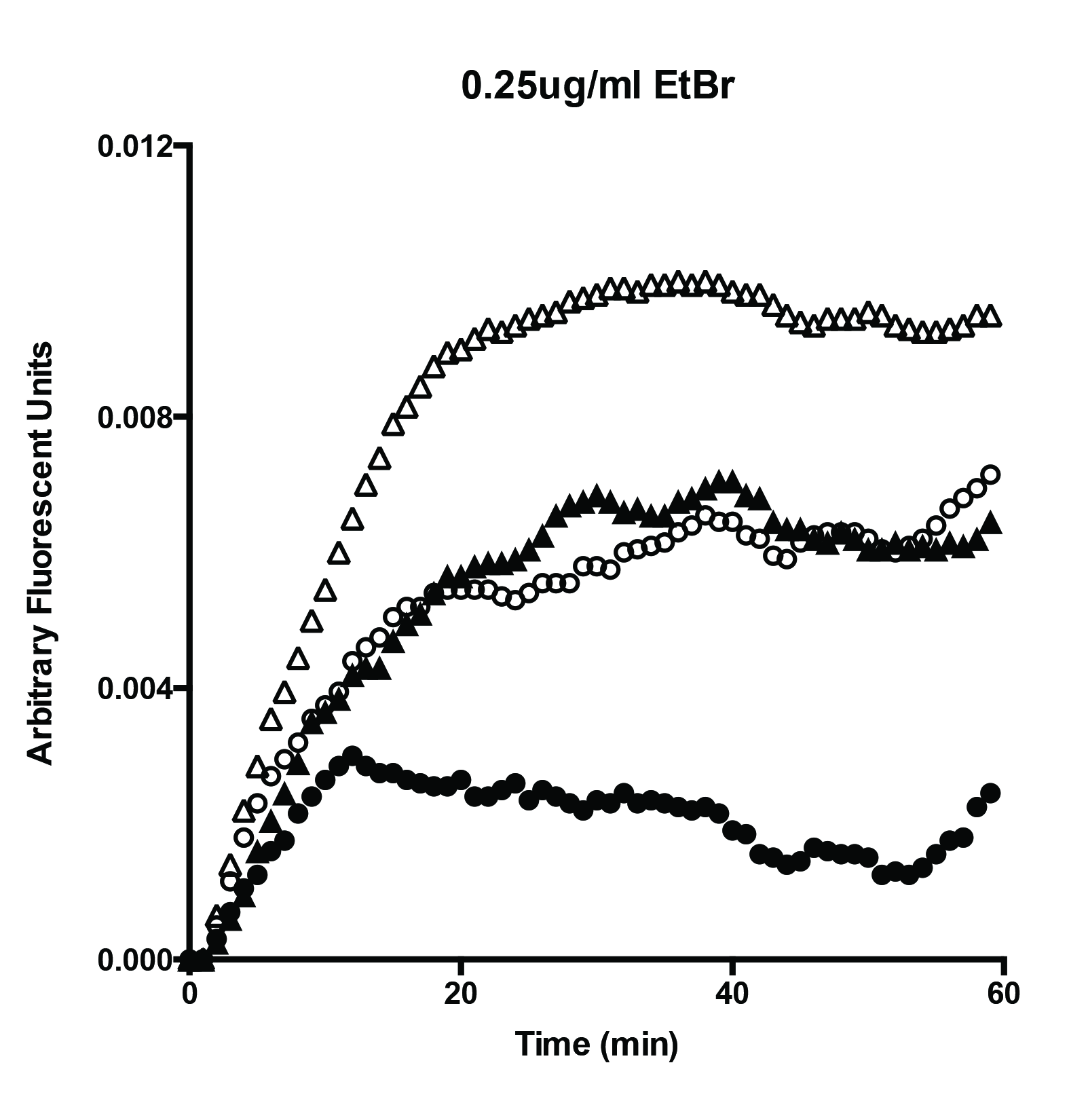

Supplement: Figure S5 — Loss of DnaK results in increased membrane permeability. Ethidium bromide uptake assay using MGM6005 18 hours after ATc withdrawal (closed triangles) or DnaK replete (closed circles). Efflux inhibitor, CCCP, added just prior to start of assay to depleted cells (open triangles) and replete cells (open circles). Time indicated at bottom as minutes after the addition of ethidium bromide. Y axis is fluorescent units calibrated for ethidium bromide (see materials and methods). (TIF) [file pgen.1004516.s005.tif]

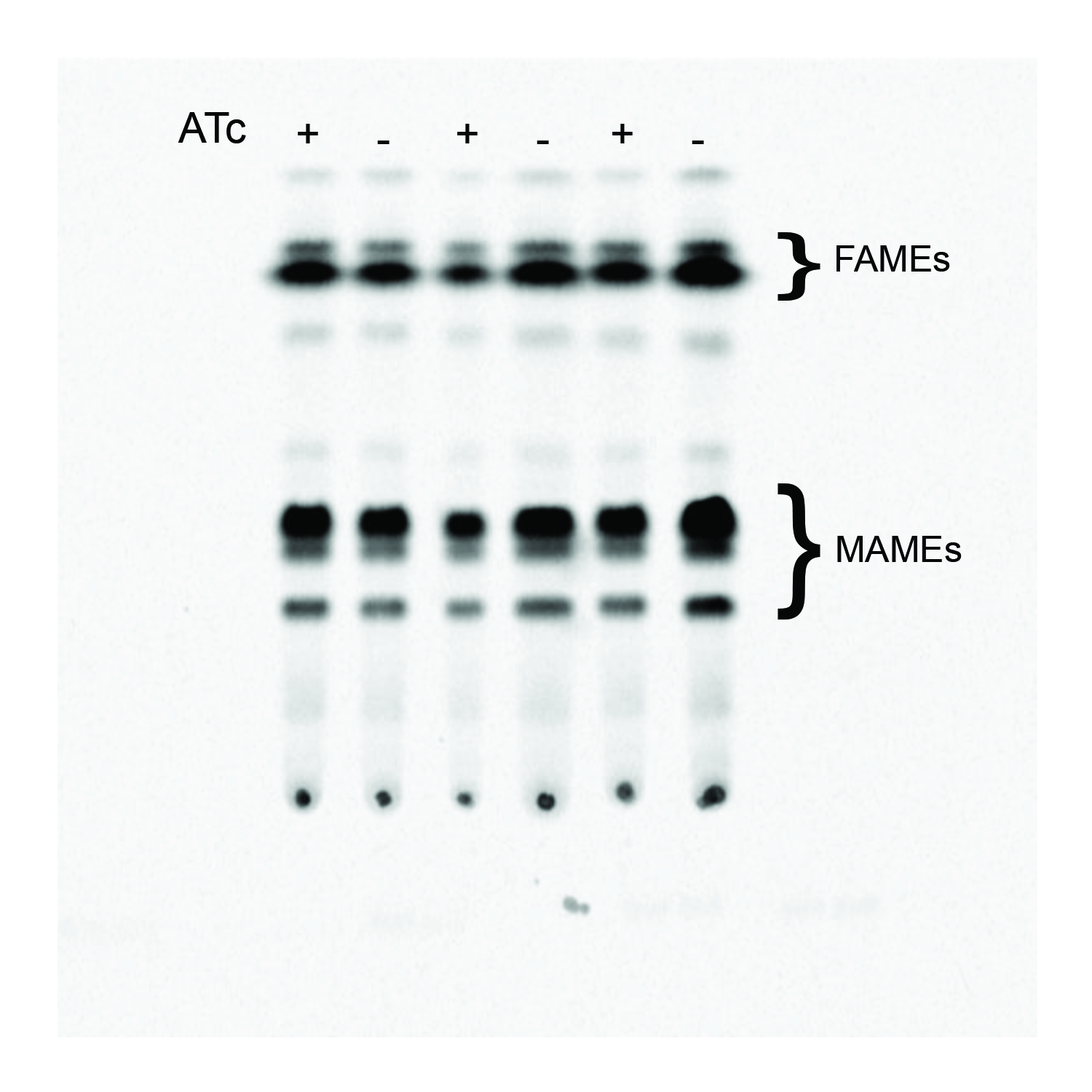

Supplement: Figure S6 — Loss of DnaK does not affect synthesis of fatty acid methyl esters (FAMEs) or mycolic acid methyl esters (MAMEs). 14C-labeled lipids from MGM6005 16 hours after ATc withdrawal to deplete DnaK (−), or with DnaK (+). Triplicate cultures are shown. (TIF) [file pgen.1004516.s006.tif]

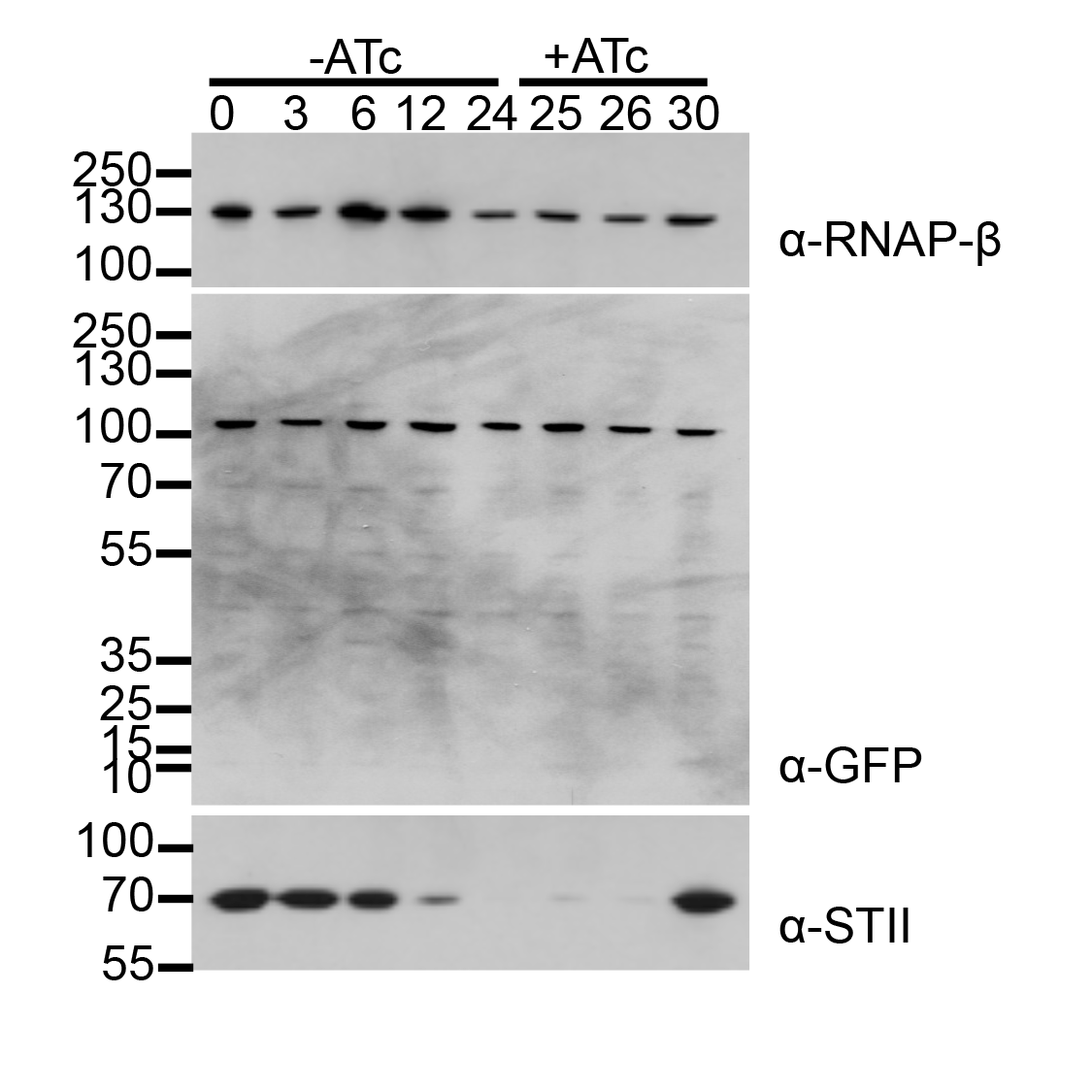

Supplement: Figure S7 — Luciferase-mCitrine protein remains stable during DnaK depletion and upon restoration of DnaK expression during outgrowth. Lysates from the Tet-DnaK, Luciferase-mCitrine expression strain (MGM6010) prepared during 24 hours of ATc withdrawal and continued through 6 hours of subsequent induction of DnaK with ATc and outgrowth. Luciferase-mCitrine runs as stable protein at estimated full length, 88 kDa, during DnaK depletion and outgrowth. Immunoblots probed for GFP (Luciferase-mCitrine, middle panel), StrepTagII (DnaK-STII, bottom panel) and RNAP-β as loading control (top panel). (TIF) [file pgen.1004516.s007.tif]

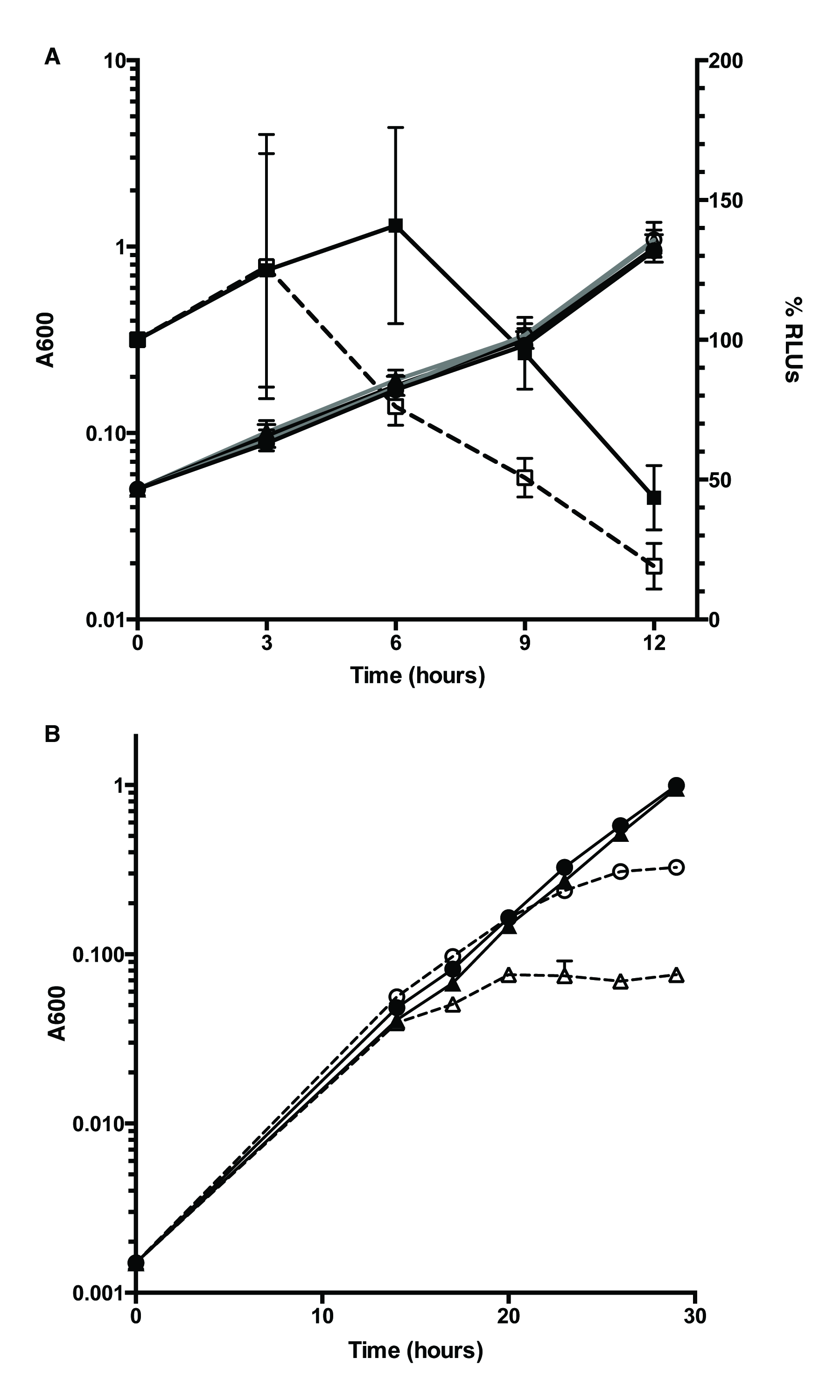

Supplement: Figure S8 — Luciferase activity and growth arrest of DnaK depletion in the presence and absence of Trigger Factor. (A) A600 and relative Luciferase activity of a Tet-DnaK strain constitutively expressing firefly luciferase, MGM6006 (+TF) and MGM6071 (−TF). A600 (plotted on left Y axis) for +DnaK/+TF indicated by closed circles/black line, for −DnaK/+TF as open circles/grey line, for +DnaK/−TF indicated by closed triangles/black line, and for −DnaK/−TF as open triangles/grey line, for +DnaK/−TF indicated by closed triangles/solid line. %RLUs (plotted on right Y axis), calculated as Counts per second (CPS) of –DnaK cultures divided by CPS of +DnaK cultures multiplied by 100, +TF indicated by closed squares/solid line and −TF indicated by open squares/dashed line. Time indicated on X axis in hours. Each point is the mean of 3 independent cultures. Error bars indicate standard deviation of replicates. (B) Growth of DnaK replete and depleted cells in the presence or absence of TF. A600 (plotted on left Y axis) for +DnaK/+TF indicated by closed circles/solid line, for −DnaK/+TF as open circles/dashed line, for +DnaK/−TF indicated by closed triangles/solid line, and for −DnaK/−TF as open triangles/dashed line, for +DnaK/−TF indicated by closed triangles/solid line. (TIF) [file pgen.1004516.s008.tif]

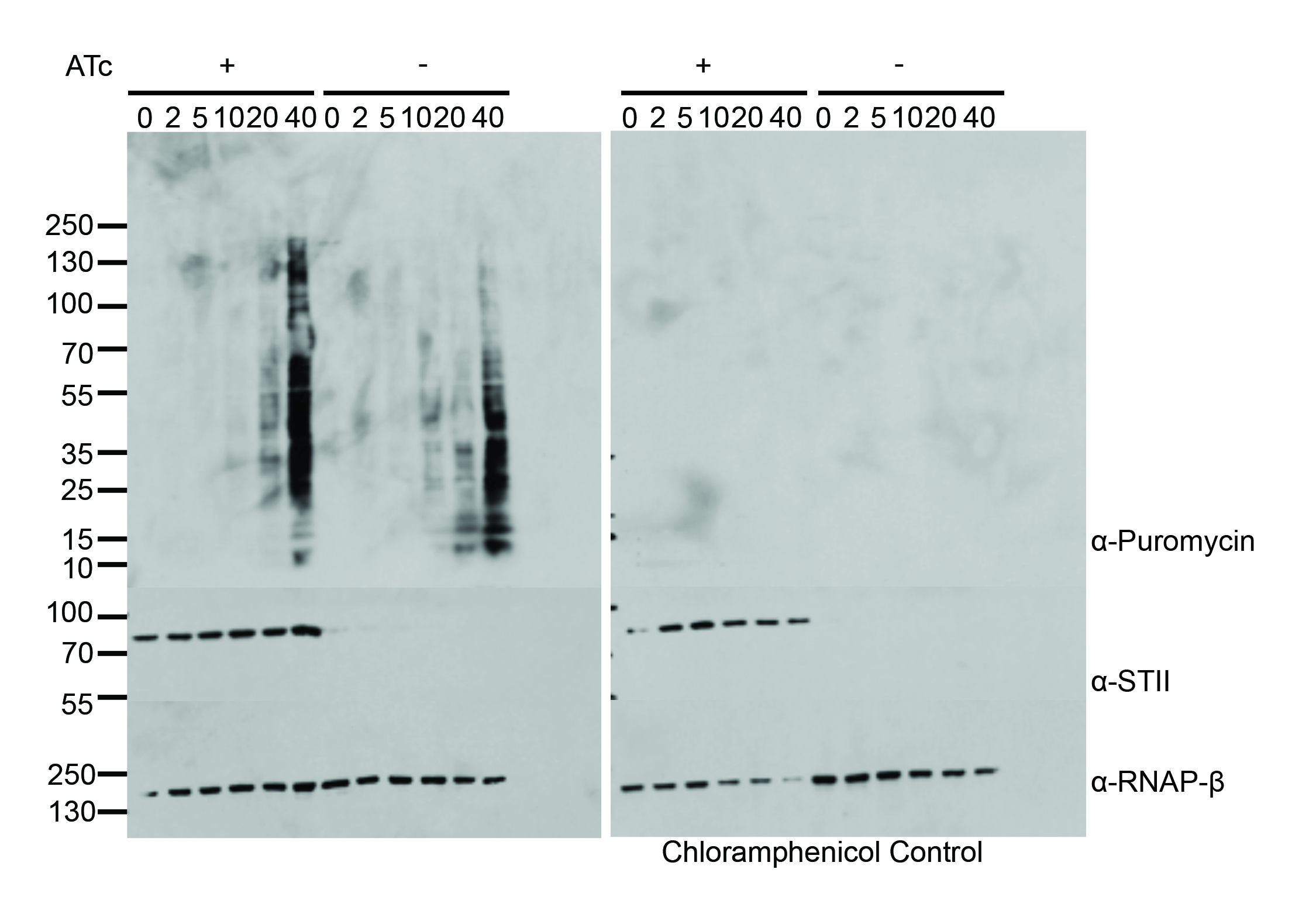

Supplement: Figure S9 — DnaK depleted and replete cells have similar translation rates. Puromycin incorporation in Tet-DnaK strain, MGM6005 started 16 hours after ATc withdrawal. Time indicated at bottom as minutes after addition of 50 µg/ml puromycin-HCl. The right panel is chloramphenicol treated controls for translation inhibition. Immunoblots probed for Puromycin (top panel), StrepTagII (DnaK-STII), and RNAP-β as loading control (bottom panel). (TIF) [file pgen.1004516.s009.tif]

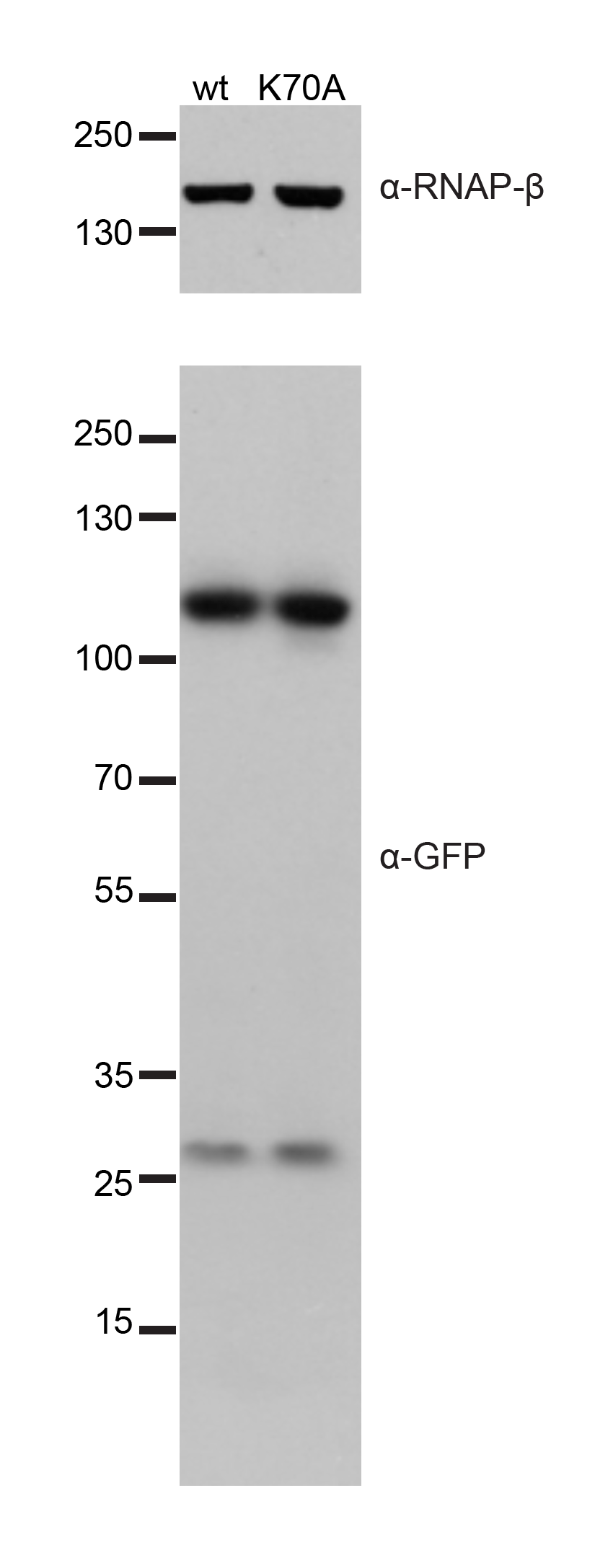

Supplement: Figure S10 — DnaK-mCitrine and DnaK(K70A)-mCitrine are expressed as stable proteins at predicted size (100 kDa). Immunoblot of lysates made from DnaK-mCitrine expression strain (MGM6003), lane 1, and DnaK(K70A)-mCitrine (MGM6024), lane 2. Immunoblots probed for GFP (DnaK-mCitrine, bottom panel) and RNAP-β as loading control (top panel). (TIF) [file pgen.1004516.s010.tif]

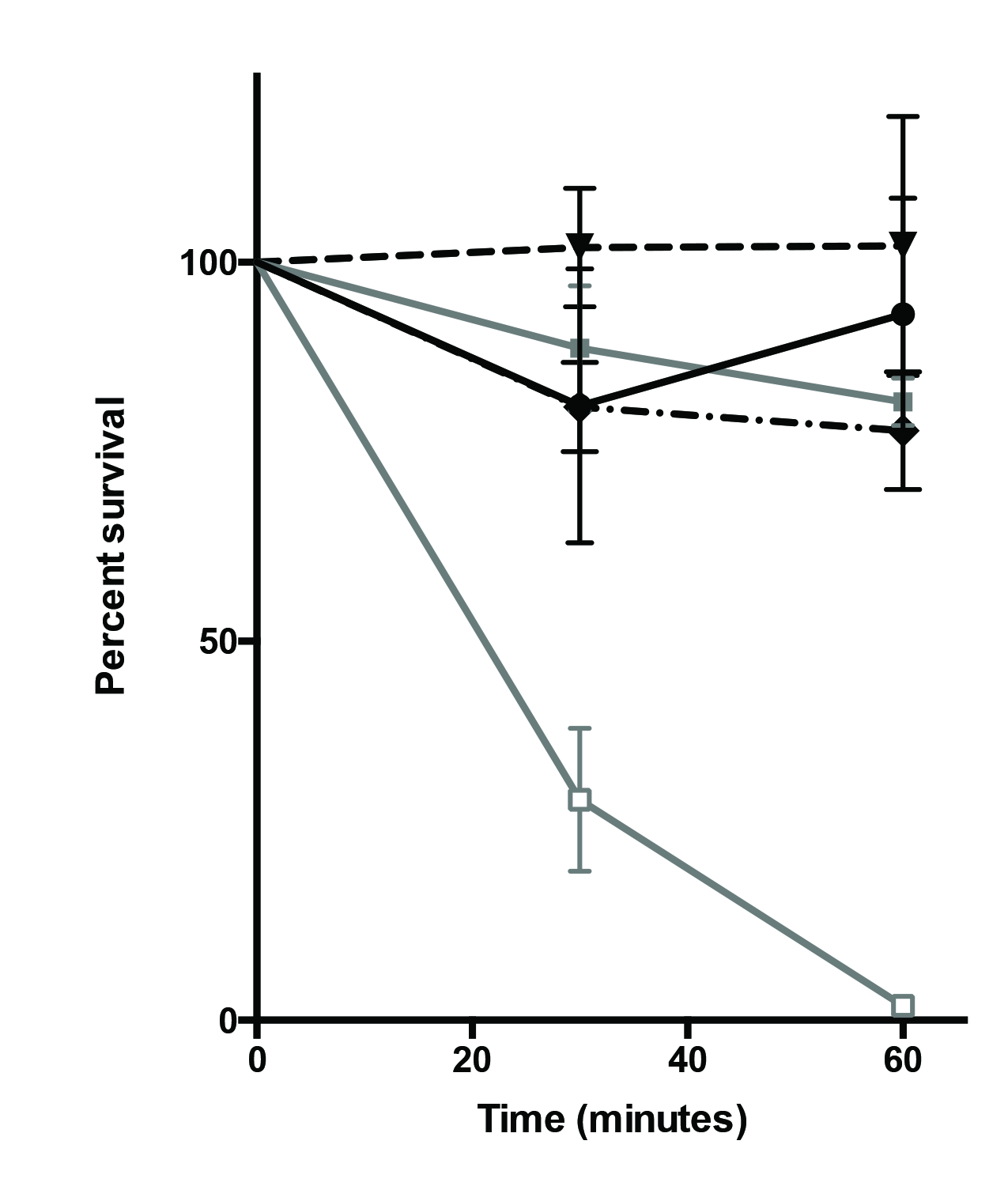

Supplement: Figure S11 — Heat sensitivity of strains expressing tagged DnaK and ClpB. Heat sensitivity of MGM6005 depleted for DnaK for 12 hours (grey open squares, dashed line) or not depleted (grey closed square, solid line), wildtype (closed circles/solid line), MGM6003 (DnaK-mCitrine, closed triangles/dashed line), and MGM6009 (ClpB-mCitrine, closed diamonds/dashed line). CFU/ml is plotted on the Y axis and time of incubation at 53°C on the X axis. (TIF) [file pgen.1004516.s011.tif]

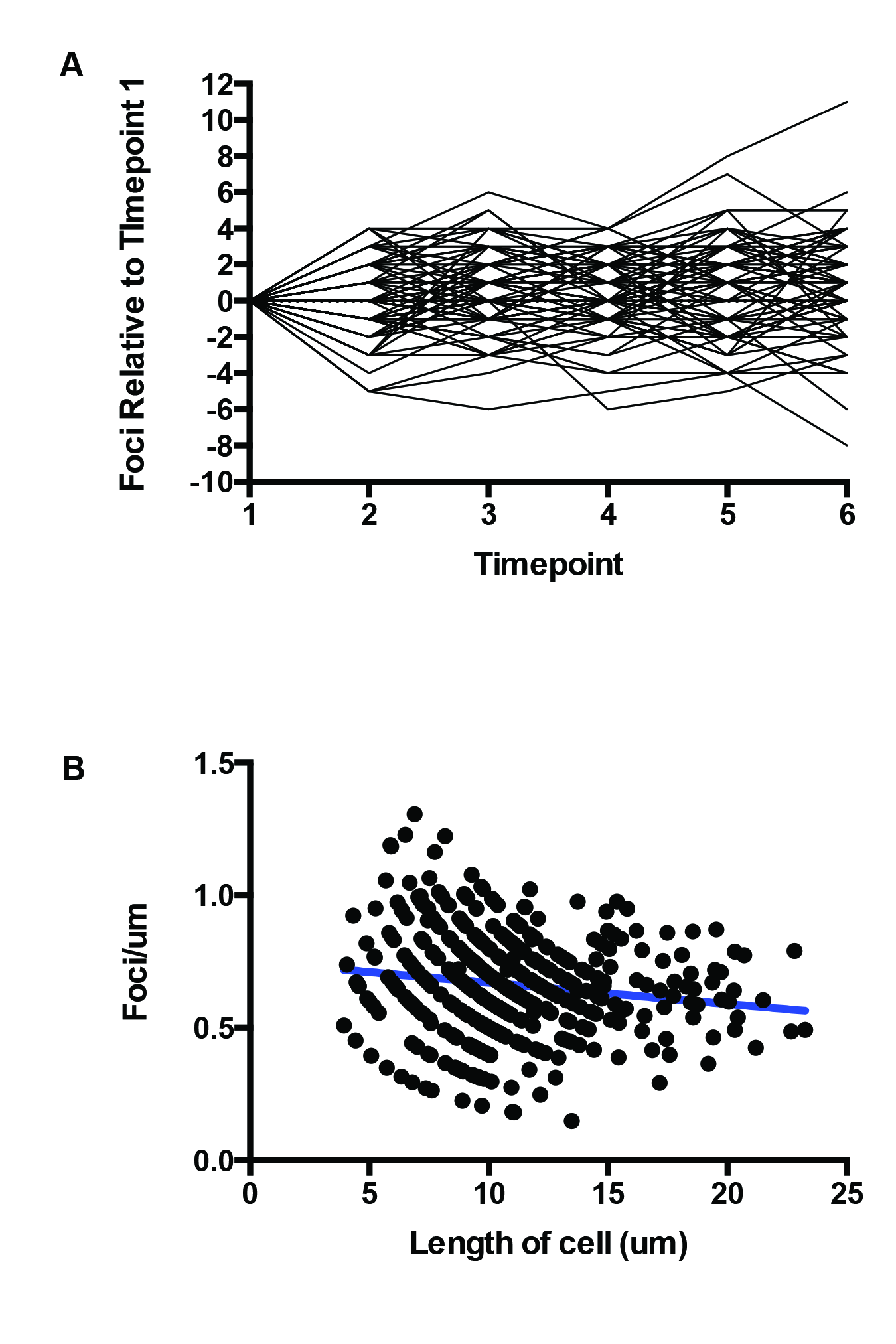

Supplement: Figure S12 — DnaK foci are dynamic in growing cells and their density does not reflect difference in cell length. The number of DnaK foci were quantitated for 76 individual cells over 6 timepoints from timelapse micrscopy images. (A) Change in foci in each cell of the 76 cells over time. Relative change in number of foci compared to timepoint 1 plotted on the Y axis for each of the 6 timepoints plotted on the X axis. (B) Foci per micron of cell length as plotted against cell length. Each of the 76 cells plotted for all 6 timepoints for a total of 456 points. Foci/µm plotted on the Y axis and length of cell plotted on the X axis. Linear regression calculated is shown with blue line. (TIF) [file pgen.1004516.s012.tif]

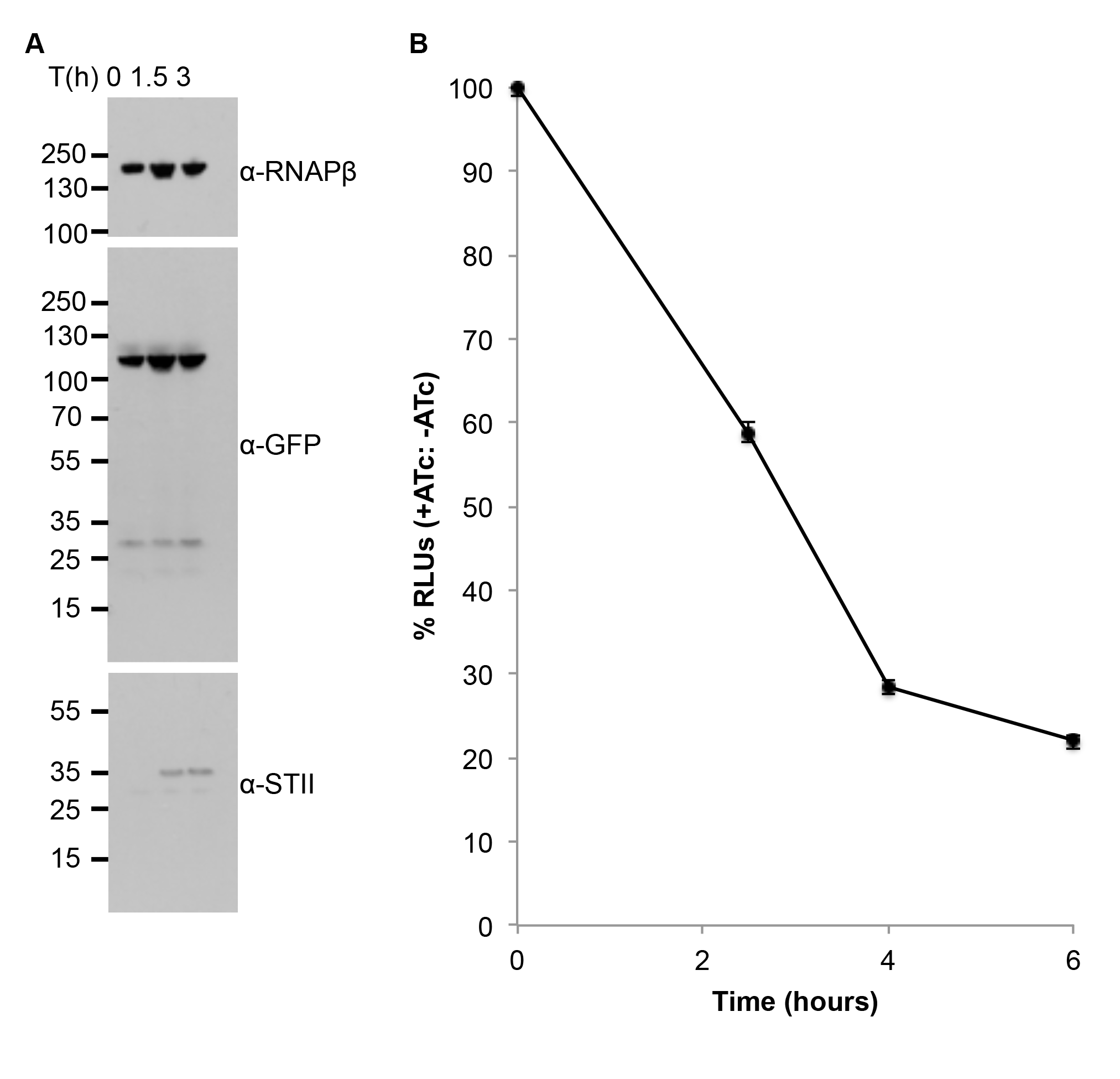

Supplement: Figure S13 — GrpE overexpression impairs DnaK-mCitrine chaperone function, but not DnaK-mCitrine protein stability. (A) Immunoblot of lysates from DnaK-mCitrine, Luciferase and Tet-GrpE expression strain (MGM6016) after 0, 1.5, and 3 hours of GrpE induction with ATc. Immunoblots probed for RNAP-β as loading control (top panel), GFP (DnaK-mCitrine, middle panel), and STII (GrpE-STII, bottom panel). (B) Luciferase activity of MGM6016 up to 6 hours after start of GrpE-STII induction with ATc. %RLUs (plotted on Y axis) calculated as (CPS (−ATc)/CPS(+ATc))*100. Time indicated on X axis in hours. Each point is the mean of 3 independent cultures. (TIF) [file pgen.1004516.s013.tif]
