# Supplementary material for: An Essential Nonredundant Role for Mycobacterial DnaK in Native Protein Folding
Source: PLoS Genet. 2014 Jul 24;10(7):e1004516. doi: 10.1371/journal.pgen.1004516 (PMC4109909; doi:10.1371/journal.pgen.1004516)
Supplement: Table S1 — Bacterial strains used in this study. Listed are the strain name, relevant genotype, source and details of construction for each strain. (PDF) [file pgen.1004516.s014.pdf]

Table S1. Strains used in this study

Strain name	*genotype*	Source	Details of construction:
MC2155	*wildtype*	[1]	
MGM6002	*ΔdnaK attB::dnaK strep*	this work	MC2155 transformed with pAJF510 and then pAJF223
MGM6003	*ΔdnaK attB::dnaK-mCitrine kan*	this work	MGM6002 transformed with pAJF200 and verified as StrepS
MGM6005	*ΔdnaK attB::kan pAJF241 (Tet-DnaK)*	this work	MGM6002 co-transformed with pAJF307 and pAJF241
MGM6006	*ΔdnaK attB::PftsZp1-Ff luciferase kan pAJF241 (Tet-DnaK)*	this work	MGM6002 co-transformed with pAJF251 and pAJF241
MGM6007	*ΔdnaK attB::dnaK strep clpB:clpB-mCitrine*	this work	MGM6002 transformed with pAJF324
MGM6008	*ΔdnaK attB::zeo clpB:clpB-mCitrine hyg pAJF327 (Tet-DnaK)*	this work	MGM6007 co-transformed with pDB19 and pAJF327
MGM6009	*clpB::clpB-mCitrine*	this work	MC2155 transformed with pAJF324
MGM6010	*ΔdnaK attB::PftsZp1-Ff luciferase-mCitrine strep pAJF241 (Tet-DnaK)*	this work	MGM6003 co-transformed with pAJF417 and pAJF241
MGM6011	*ΔdnaK attB::dnaK-mCitrine kan pAJF488 (Tet-DnaK)*	this work	MGM6003 transformed with pAJF488
MGM6012	*clpB:clpB-mCitrine pAJF494 (Tet-mCerulean-ELK16)*	this work	MGM6009 transformed with pAJF494
MGM6013	*ΔdnaK attB::dnaK strep fasI:fasI-mCitrine hyg*	this work	MGM6002 transformed with pAJF438
MGM6014	*ΔdnaK attB::zeo fasI:fasI-mCitrine hyg pAJF327 (Tet-DnaK)*	this work	MGM6013 co-transformed with pDB19 and pAJF327
MGM6015	*ΔdnaK attB::zeo pAJF458 (Tet-DnaK EcmalF(1,2)-3xmCerulean)*	this work	MGM6002 co-transformed with pDB19 and pAJF458
MGM6016	*ΔdnaK attB::dnaK-mCitrine kan* $attB_{tweety}$*::PftsZ1-Ff luciferase strep pAJF499 (Tet-GrpE)*	this work	MGM6003 co-transformed with pAJF381 and pAJF499
MGM6022	*pAJF480 (Tet-mCitrine-ELK16)*	this work	MC2155 transformed with pAJF480
MGM6023	*Mycobacterium bovis BCG att::dnaK-mCitrine kan*	this work	*M. bovis BCG pasteur* (ATCC #35734*)* transformed with pAJF466
MGM6024	*attB::dnaK(K70A)-mCitrine kan*	this work	MC2155 transformed with pAJF239

MGM6027	*ΔgrpE attB::PdnaK-grpE strep*	this work	MC2155 transformed with pAJF325 and then pAJF512
MGM6028	Δ*tig*	this work	MC2155 transformed with pAJF273
MGM6031	Δtig ΔdnaK attB::dnaK-mCitrine kan	this work	ΔdnaK added with pAJF510
MGM6052	*Δtig ΔdnaK attB::dnaK strep*	this work	MGM6031 transformed with pAJF223
MGM6071	*Δtig ΔdnaK attB::PftsZp1-luciferase kan pAJF241 (tetR Pmyc-tetO-dnaK-STII hyg)*	this work	MGM6052 co transformed with pAJF251 and pAJF241
MGM6072	*ΔdnaK attB::dnaK strep pAJF407 (tetR Pmyc-tetO-tig kan)*	this work	MGM6002 transformed with pAJF407
MGM6073	*ΔdnaK attB:: PftsZp1-luciferase Pmop-tig kan pAJF241 (tetR Pmyc-tetO-dnaK-STII hyg)*	this work	MGM6002 co-transformed with pAJF251 and pAJF298

1. Snapper SB, Melton RE, Mustafa S, Kieser T, Jacobs WR, Jr. (1990) Isolation and characterization of efficient plasmid transformation mutants of Mycobacterium smegmatis. Mol Microbiol 4: 1911-1919.
