# Supplementary material for: An Essential Nonredundant Role for Mycobacterial DnaK in Native Protein Folding
Source: PLoS Genet. 2014 Jul 24;10(7):e1004516. doi: 10.1371/journal.pgen.1004516 (PMC4109909; doi:10.1371/journal.pgen.1004516)
Supplement: Table S2 — Plasmids used in this study. Listed are the plasmid name, relevant features, and details of construction for each plasmid used. (PDF) [file pgen.1004516.s015.pdf]

Table S2. Plasmids used in this study

Plasmid name	relevant features	relevant details of construction
pMV306hsp+FFluc	*attP::P_{hsp60}-Ff luciferase kan*	Addgene plasmid #26156 [1]
Cerulean	*mCerulean*	Addgene plasmid #15214 [2
pDB19	*attP::zeo*	lab stock
pDB60	*attP::strep*	lab stock
pDB88	*hyg, galK, sacB*	lab stock [3]
pMV306kan	*attP::kan*	lab stock
pMV261kan	*P_{hsp60} kan oriM*	lab stock
pmsg419	*P_{Tb21}-tetR(sB) $P_{myc1\text{-}tetO}$ hyg oriM*	lab stock
pAJF067	*hyg, galK, sacB*	pDB88 derivative
pAJF198	*5'dnak flank-3' dnaK flank hyg, galK, sacB*	pAJF067 with *dnaK* 5' flank (oAF252/oAF253, MC2155 gDNA) dnaK 3' flank (oAF254/oAF255, MC2155 gDNA)
pAJF200	*attP:: dnaK-mCitrine kan*	pMV306kan derivative with dnaK (oAF250/251, MC2155 gDNA) and mCitrine (oAF022/oAF023)
pAJF223	*attP:: dnaK strep*	pDB60 derivative with dnaK (oAF250/266, MC2155 gDNA)
pAJF239	*attP:: dnaK(K70A)-mCitrine kan*	pAJF200 with 5' dnaK(K70A) (flanks of oAF250/oAF301 and oAF276/oAF302 in pAJF200)
pAJF241	*P_{Tb21}-tetR(sB) $P_{myc1\text{-}tetO}$-dnaK-STII hyg oriM*	pmsg419 with dnaK-streptagII (oAF305/oAF280, MC2155 gDNA)
pAJF251	*attP::$P_{ftsZ(P1)}$-Ff luciferase kan*	pMV306-hsp+FFLuc with PftsW(P1) (oAF312/313, MC2155 gDNA)
pAJF273	*5'tig flank-3' tig flank hyg, galK, sacB*	pAJF067 derivative with tig 5' flank (oAF347/oAF348, MC2155 gDNA) and tig 3' flank (oAF349/oAF350, MC2155 gDNA)
pAJF298	*attB:: PftsZp1-luciferase Pmop-tig kan*	pAJF251 derivative with tig (oAF336/oAF337, MC2155 gDNA)
pAJF307	*pMV306Kan No Integrase*	pMV306kan derivative with integrase sequence removed
pAJF324	*3'clpB-mCitrine hyg*	3' clpB (oAF400/oAF401, MC2155 gDNA)

pAJF325	*5'grpE flank-3' grpE flank hyg, galK, sacB*	pAJF067 derivative with grpE 5' flank (oAF412/oAF413, MC2155 gDNA) and grpE 3' flank (oAF414/oAF415, MC2155 gDNA)
pAJF327	*P_{Tb21}-tetR(sB) $P_{myc1\text{-}tetO}$-dnaK-STII kan oriM*	derivative of pAJF241 encoding kanamycin resistance
pAJF381	*attP(tweety)::$P_{ftsZ(P1)}$-Ff luciferase int(tweety) strep*	PftsZ1-Ff luciferase fragment from pAJF251 inserted at EcoRV site
pAJF407	*P_{Tb21}-tetR(sB) $P_{myc1\text{-}tetO}$-dnaK-STII kan oriM*	pAJF327 derivative with tig (oAF575/oAF576, MC2155 gDNA)
pAJF417	*attP::$P_{ftsZ(P1)}$-Ff luciferase-linker-mCitrine strep*	pDB60 derivative with PftsZ(P1)-Ff luciferase from oAJF251 fused to mCitrine
pAJF438	*3'fas-linker-mCitrine-STII hyg*	3' fasI (oAF642/oAF643, MC2155 gDNA)
pAJF446	*P_{hsp60}-Ec $maL_{(TM1,2)}$-mCerulean-linker-mCerulean- linker-mCerulean kan*	pMV261kan derivative with E. coli $malF_{(TM1,2)}$ (oAF578/579, MG1665 gDNA)
pAJF447	*attP:: dnaK-twinStrep kan*	pAJF200 with mCitrine removed and twinStrep added by oligo ligation (oAF651/oAF652)
pAJF458	*P_{Tb21}-tetR(sB) $P_{myc1\text{-}tetO}$-dnaK-STII hyg oriM Phsp60-EcmalF1,2-3xmCerulean*	pAJF241 derivative with Phsp60-EcmalF1,2-3xmCerulean cut from pAJF446
pAJF466	*attB::BCG dnaK-mCitrine strep*	BCG dnaK (oAF674/oAF676, BCG gDNA)
pAJF480	*P_{Tb21}-tetR(sB) $P_{myc\text{-}tetO}$-mCitrine-ELK16 hyg oriM*	pmsg419 derivative with mCitrine-ELK16 (tag added with ligation of annealed oligos oAF708 and oAF709)
pAJF488	*P_{Tb21}-tetR(sB) $P_{myc1\text{-}tetO}$-mCerulean-ELK16 hyg oriM*	pmsg419 derivative with mCerulean-ELK16 (tag added with ligation of annealed oligos oAF708 and oAF709)
pAJF494	*P_{Tb21}-tetR(sB) $P_{myc1\text{-}tetO}$-mCerulean-ELK16 strep oriM*	pAJF488 derivative encoding streptomycin resistance
pAJF499	*P_{Tb21}-tetR(sB), $P_{myc1\text{-}tetO}$-grpE-STII, hyg, oriM*	pmsg419 derivative with grpE-STII (oAF469/470, MC2155 gDNA)
pAJF509	*attP::P_{dnaK}-grpE kan*	pMV306kan derivative with PdnaK (oAF330/oAF331, MC2155 gDNA) and grpE (oAF762/764, MC2155 gDNA)
pAJF512	*attP::P_{dnaK}-grpE strep*	pDB60 with PdnaK-grpE from pAJF509

1. Andreu N, Zelmer A, Fletcher T, Elkington PT, Ward TH, et al. (2010) Optimisation of bioluminescent reporters for use with mycobacteria. PLoS One 5: e10777.

2 Rizzo MA, Springer GH, Granada B, Piston DW (2004) An improved cyan fluorescent protein variant useful for FRET. Nat Biotechnol 22: 445-449.
3. Barkan D, Stallings CL, Glickman MS (2011) An improved counterselectable marker system for mycobacterial recombination using galK and 2-deoxy-galactose. Gene 470: 31-36.
