# Supplementary material for: An Essential Nonredundant Role for Mycobacterial DnaK in Native Protein Folding
Source: PLoS Genet. 2014 Jul 24;10(7):e1004516. doi: 10.1371/journal.pgen.1004516 (PMC4109909; doi:10.1371/journal.pgen.1004516)
Supplement: Table S3 — Oligonucleotides used in this study. Listed are the oligo name, sequence, and (for qPCR oligos) the target gene amplified. (PDF) [file pgen.1004516.s016.pdf]

Table S3. Oligos used in this study.

A.

Oligos for qPCR

Oligo Name	5'-3' sequence	*gene*
oAF485	CCAAGCGGGCAGCCAAG	*sigA*
oAF486	GGGCTCAGCCTCGAGATCGTC	*sigA*
oAF473	CGCTGGCAACCCGGAGATC	*clpB*
oAF474	TGGGCAGACGGTCGAGCAA	*clpB*
oAF475	CACCGAGCTTGAGCCCGACTT	*tig*
oAF476	GGGCAGCGCGTCGTTGA	*tig*
oAF496	CGAGGACCCCTACGAGAAGAT	*groL1*
oAF497	GGGTTGGCGCCGGCAGC	*groL1*
oAF502	GGTGCTGGCCAAGTCTTTCGG	*groL2*
oAF503	CACCGGCGACGTCGTTGG	*groL2*
oAF500	GCACATCCTTTTGGGACTTATTCA	*clpC*
oAF501	CAGCTCGAGCACCTTCTTGGC	*clpC*
oAF508	ACGTTTCTGATCTCTGTGGC	*hspR*
oAF509	AGCCGCTGCACCTCTCGC	*hspR*
oAF518	ACGCCCGCGGTGTGGAC	*hsp20*
oAF519	CCACACCGGGGAGTTCG	*hsp20*

B.

Oligos used for plasmid construction

Oligo Name	5'-3' sequence
oAF022	GGCAGATCTCATATGATGGTGAGCAAGGGCG
oAF023	GATCAAGCTTTTACTTGTACAGCTCGTC
oAF250	GAATCTAGAAGAAGAACCTGGTGG
oAF251	GATCATATGCTTGTTCTCCTTGCC
oAF252	GATACTAGTCCGGTCCGCGGAAAC
oAF253	GTTCATATGGTTCTTGCCTCCTGATAG
oAF255	GTTCCTAGGCCCCAGAGCCGTCCTC
oAF280	GTCAATTGTTACTTTTCGAACTGCGGGTGGCTCCACTTGTTCTCCTTGCC
oAF301	CACCATCCGTTCGGTCGCGCGCCACGTCGGCAC
oAF302	GTGCCGACGTGGCGCGCGACCGAACGGATGGTG
oAF305	GTTGCATGCCCTATATCTATCAGG
oAF312	GTTGCGGCCGCTACCGTTCTGTTTGC
oAF313	GTCGAATTCGGTTGGGTGCTCTGG
oAF330	GATTCTAGACATGGCGATGCGTGAAC
oAF331	GTTCCATGGTCTTGCCTCCTGATAG

oAF336	GTTCATATGGACACCGAGAAAGACATC
oAF337	GTTATCGATTCACTTGTCGGTGTCG
oAF347	GTTACTAGTGTAGTAGCCAGGCGGATAC
oAF348	GATCATATGCTTGTGCTCCTTGATG
oAF349	GATCATATGCGCTGTGAGCGAAC
oAF350	GTTCCTAGGGAATCGGCCTCGATG
oAF400	GTATGCGCATGGAGATCG
oAF401	GTCCATATGGCCCAAAACGAGGGAC
oAF412	GTTACTAGTCTCGCACAACAAGCTG
oAF413	GTTCATATGTTGTTCTCCTTGCCAG
oAF414	GTTCATATGACAGATCCGACTAGAAAG
oAF415	GTTCCTAGGTGATCACACCGGAGCC
oAF469	GTTGCATGCACGATGACGCTGGCAAG
oAF470	GTCAAGCTTACTTTTCGAACTGCGGGTGGCTCCAATTGTCTGATTCTGC
oAF575	GTTATCGATGTGAAGAGCACAGTCG
oAF576	GATAACGTTACTTGTCGGTGTCGTCG
oAF478	GGATCTCCGCGGGTTTGGG
oAF578	GCCTGGCCAGCGATGTCATTAAAAAG
oAF579	GACCATATGGGCTTTACGATTGGC
oAF642	GATTCTAGAAAGCACGACACCTCG
oAF643	GTTCATATGCAGGACGTACTGATC
oAF651	TATGTCAGCATGGAGCCACCCGCAGTTCGAAAAGGGCGGTGGATCAGGCGGCGGATCCGGAGGATC AGCATGGAGTCATCCACAATTCGAGAAGTA
oAF652	AGCTTACTTCTCGAATTGTGGATGACTCCATGCTGATCCTCCGGATCCGCCGCCTGATCCACCGCC CTTTTCGAACTGCGGGTGGCTCCATGCTGACA
oAF674	GAATCTAGAATCGAGGAGTGGGTG
oAF676	GTTCATATGCTTGGCCTCCCGGCCGTC
oAF708	AATTCCTCGAGCTGGAACTGAAGTTGAAGCTCGAGCTGGAGCTGAAACTCAAGTA
oAF709	AGCTTACTTGAGTTTCAGCTCCAGCTCGAGCTTCAACTTCAGTTCCAGCTCGAGG
oAF762	GCGCAGTCCGCTCAGAACGATGACGCTGGCAAG
oAF764	CGTCGACATCGATAAGCTTAATTGTCTGATTCTGC
oAF768	GAACATATGAGGCCGAACAGCCCGCG
